# Observed positive vegetation-rainfall feedbacks in the Sahel dominated by a moisture recycling mechanism

**DOI:** 10.1038/s41467-017-02021-1

**Published:** 2017-11-30

**Authors:** Yan Yu, Michael Notaro, Fuyao Wang, Jiafu Mao, Xiaoying Shi, Yaxing Wei

**Affiliations:** 1 Nelson Institute Center for Climatic Research, University of Wisconsin-Madison, Madison, WI 53706 USA; 2 Jet Propulsion Laboratory, California Institute of Technology, Pasadena, CA 91109 USA; 3 Environmental Sciences Division and Climate Change Science Institute, Oak Ridge National Laboratory, Oak Ridge, TN 37831 USA

## Abstract

Classic, model-based theory of land-atmosphere interactions across the Sahel promote positive vegetation-rainfall feedbacks dominated by surface albedo mechanism. However, neither the proposed positive vegetation-rainfall feedback nor its underlying albedo mechanism has been convincingly demonstrated using observational data. Here, we present observational evidence for the region’s proposed positive vegetation-rainfall feedback on the seasonal to interannual time scale, and find that it is associated with a moisture recycling mechanism, rather than the classic albedo-based mechanism. Positive anomalies of remotely sensed vegetation greenness across the Sahel during the late and post-monsoon periods favor enhanced evapotranspiration, precipitable water, convective activity and rainfall, indicative of amplified moisture recycling. The identified modest low-level cooling and anomalous atmospheric subsidence in response to positive vegetation greenness anomalies are counter to the responses expected through the classic vegetation-albedo feedback mechanism. The observational analysis further reveals enhanced dust emissions in response to diminished Sahel vegetation growth, potentially contributing to the positive vegetation-rainfall feedback.

## Abstract (web summary)

Vegetation-rainfall feedbacks in the Sahel are thought to be positive, but the precise mechanisms for this are unclear. Here the authors analyse observations to show that a moisture recycling mechanism drives this feedback.

## Introduction

The Sahel is characterized by substantial interannual to decadal variability in rainfall and high socio-economic vulnerability to hydrologic extremes^1^. Attribution of this pronounced rainfall variability to either oceanic^3–5^ or terrestrial^6–13^ drivers has proven to be an elusive challenge. The Sahel experienced one of the most extreme and prolonged droughts in the global observational record during the 1970s-1990s^2, 14, 15^, which was believed to be mainly driven by external forcing from sea-surface temperature (SST) anomalies and amplified by local feedbacks associated with land degradation and desertification according to past modeling studies^1, 5, 10, 12, 16–18^. Despite vegetation’s apparent significant role in amplifying and extending this drought episode^10, 12^, the current understanding of vegetation’s impacts on climate across the Sahel, through proposed albedo, moisture, and momentum feedbacks, originates from biased and highly parameterized climate models, with land-atmosphere interactions that remain insufficiently tested against observations. Charney^6^ first hypothesized a positive vegetation-rainfall feedback regarding Sahel desertification, in which reduced greenness leads to increased surface albedo, resulting in low-level cooling, increased atmospheric stability, low-level subsidence, and drying. Other modeling studies have also simulated this proposed positive feedback but disagreed in terms of the relative dominance of the albedo and moisture mechanisms^10, 12, 13^, likely due to the exaggerated albedo forcing applied in the earlier modeling study^6^.

Most studies on biophysical vegetation feedbacks have been restricted to running and analyzing coupled vegetation-climate model simulations, which have several key limitations^19^. Simulated feedbacks are model dependent, given that climate models vary in terms of dynamical core, numerical schemes, parameterizations, and spatial resolution^20^. Many modeling studies have applied extreme sensitivity experiments, such as complete, instantaneous regional deforestation, which have limited real-world relevance, as observed vegetation changes are typically heterogeneous in space and transient in time. Owing to these modeling study limitations, observational studies of vegetation feedbacks are critically needed for testing the model-based hypotheses^21^ but would have to address several key challenges. First, observational records are generally short in duration and marred by uncertainty from measurement errors. Second, it is challenging to extract the observed signal of vegetation forcing on the atmosphere from the large atmospheric internal noise, especially given that the atmospheric forcing on vegetation clearly outweighs vegetation’s feedback to the atmosphere^20, 22^. Third, regional climate is affected by variability in slowly-evolving forcings from both the oceans and vegetation, making it difficult to distinctly separate their individual contributions through traditional regression-based analyses.

Previous observational studies on vegetation feedbacks across the Sahel are largely based on multiple linear regression and apply a single observational or reanalysis product, leading to limited credibility in their conclusions and no quantification of uncertainty. Evidence of positive vegetation-rainfall feedbacks has been found from previous observational studies using a statistical vegetation index simulation^23^ and Granger causality analysis^24–26^. However, these observational investigations, based on multiple linear regression, do not fully tease out potential oceanic impacts on the variability in both the vegetation and precipitation, either due to the absence of oceanic predictors in their analysis or limitations of the applied methodology itself, namely that multiple linear regression-based methods are unable to address highly correlated predictors, thereby biasing the assessed terrestrial impacts. Furthermore, their analyses are generally based on a single observational or reanalysis product for each variable, e.g., precipitation from the Climatic Research Unit (CRU), which has limited gauge coverage and thus limited reliability in the Sahel. Moreover, none of the previous observational studies have quantified the influence of observational uncertainty on the identified vegetation-rainfall feedbacks. Therefore, both the observed positive vegetation-rainfall feedback and the underlying mechanisms need more sophisticated and comprehensive investigations.

The apparent recovery from the multi-decadal drought during the early 21st century and the underlying recovery mechanism remain highly debated, partly due to increased interannual variability in Sahel rainfall^27^. In particular, the recent interannual variability in Sahel rainfall was not successfully predicted by the SST forcings that explained the late 20th century decadal drought in most state-of-the-art climate models, implying either recent changes in oceanic drivers of the interannual Sahel rainfall variability or elevated importance of other regulators, including land surface feedbacks^28^. Despite this model-based hypothesis regarding the change in oceanic regulations, there has never been rigorous observational quantification of the relative contribution from oceanic versus terrestrial drivers of the recent interannual variability in Sahel rainfall. Although the multiple regression-based study attempted to quantify the relative contribution of variability in Atlantic SST and Sahel Normalized Difference Vegetation Index (NDVI) to Sahel rainfall variability^25^, the results are uncertain due to the limited reliability of multiple regression in separating impacts from individual forcings and are not directly comparable with previous modeling studies due to the absence of remote oceanic impacts assessed in their study.

A multivariate, lagged covariance statistical method, the Generalized Equilibrium Feedback Assessment (GEFA) (Methods), was developed to address the aforementioned challenges in the observational analysis of the oceanic and terrestrial forcings on the atmosphere^19, 29, 30^. GEFA’s reliability at extracting key oceanic and terrestrial drivers on North African climate, even with short data records, was successfully demonstrated through dynamical experiments with modified regional SST or leaf area index (LAI) using the National Center for Atmospheric Research (NCAR) Community Earth System Model (CESM)^31, 32^. Furthermore, it was shown that GEFA, when applied to assess land surface feedbacks, captures the combined effects of coupled vegetation and soil moisture anomalies^32^. In order to quantify uncertainty in estimated observed terrestrial feedbacks, GEFA is applied here to a spectrum of gridded observations, remote sensing products, and reanalyses, while weighting different datasets according to their estimated regional reliability across the Sahel in order to minimize the impacts of measurement errors (Methods). This multi-data set approach is particularly valuable over the data-sparse Sahel.

On the basis of GEFA as applied to multiple observational data sets, we identify positive vegetation-rainfall feedbacks across the Sahel during the late to post-monsoon periods associated with a moisture recycling mechanism. The classic albedo-based mechanism is not supported by the observational data. We further reveal that diminished vegetation growth and accompanying dry soils lead to enhanced dust emissions across the Sahel, which potentially contributes to the positive vegetation-rainfall feedback.

## Results

### Terrestrial versus oceanic contributions to Sahel climate

**Fig. 1:**
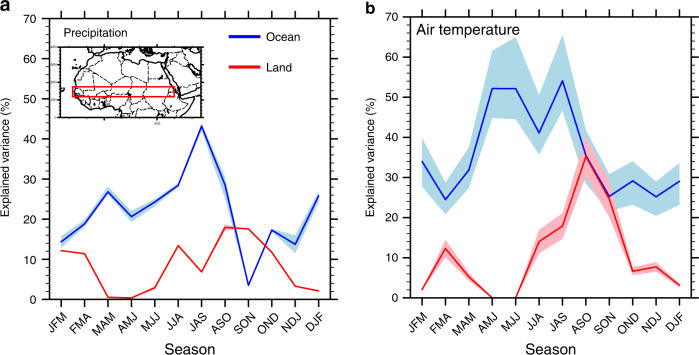
Total percent variance in observed atmospheric conditions across the Sahel explained by oceanic versus terrestrial forcings in each season during 1982–2011. **a** precipitation and **b** 2-m air temperature. The lines (red: terrestrial forcings, blue: oceanic forcings) represent the multi-data set average, and the shading represents the 10th and 90th percentiles of the multi-data set uncertainty range. Labels on the *x* axis stand for 3-month seasons, e.g., JFM for January, February, and March. The inserted map in a shows the Sahel region (12˚ N–17˚ N, 20˚ W–40˚ E). The smaller multi-data set uncertainty range in precipitation is apparently due to a greater number of datasets included in the analysis, compared to the temperature analysis. By randomly selecting two out of the four precipitation datasets, the typical multi-data set uncertainty largely increases and is comparable with that of air temperature

According to GEFA, oceanic forcings largely overwhelm terrestrial forcings on the observed variability in the Sahel’s regional precipitation and air temperature, with the exception of the post-monsoon season (SON), when vegetation feedbacks on local precipitation appear to dominate (Fig. 1). The multi-data set, average percent variance of Sahel precipitation explained by oceanic forcings is 22% on the annual mean (mainly from tropical Pacific and tropical Atlantic SSTs), ranging from 4% in September–November (SON) to 44% in July–September (JAS). Oceanic forcings impose a greater regulation on Sahel air temperature, explaining 36% of the total variance on the annual mean and ranging from 26% in SON to 53% in JAS. The explained variance in precipitation by terrestrial forcings is 8% on the annual mean and ranges from 0% in April-June (AMJ) to 18% in August-October (ASO) and SON, which is comparable in magnitude with their regulation of air temperature. SON is the only season in which the land surface forcings dominate over oceanic forcings, with land surface variability explaining 17.5–18.2% among datasets of the total variance in precipitation. Indeed, Sahel rainfall variability during SON of 1982–2010 is largely successfully reconstructed by the GEFA-based prediction model containing only terrestrial NDVI forcings, with a temporal correlation of 0.79 between the multi-data set average predicted and observed time series, compared with that of 0.81 using both SST and NDVI forcings, and 0.56 using only SST forcings (Supplementary Fig. 1b). In contrast to the late-to-post monsoon season and consistent with previous model-based findings^10, 12^, the monsoon rainfall over the Sahel during June–August is mostly regulated by oceanic drivers with a temporal correlation between the predicted and observed rainfall of 0.84 using only oceanic forcings, compare with 0.88 using both oceanic and terrestrial forcings (Supplementary Fig. 1a).

The contribution from each oceanic and terrestrial forcing on individual drought and pluvial cases can be decomposed by GEFA. For example, during 1984 (Supplementary Fig. 2a, b), which was the driest year in the Sahel during 1982–2011, an anomalously dry monsoon was favored by the Atlantic and Indian SST anomalies, as well as anomalously sparse vegetation cover in the Sahel, West African monsoon region, and Horn of Africa. This drought continued during the post-monsoon season, mainly driven by the anomalies in Sahel vegetation cover. As another example, during 1999 (Supplementary Fig. 2c, d), which was one of the wettest years during the study period, an anomalously wet Sahel monsoon was mainly supported by La Niña conditions, and extended into the post-monsoon season, when oceanic impacts largely diminished and terrestrial forcings were allowed to dominate.

**Fig. 2:**
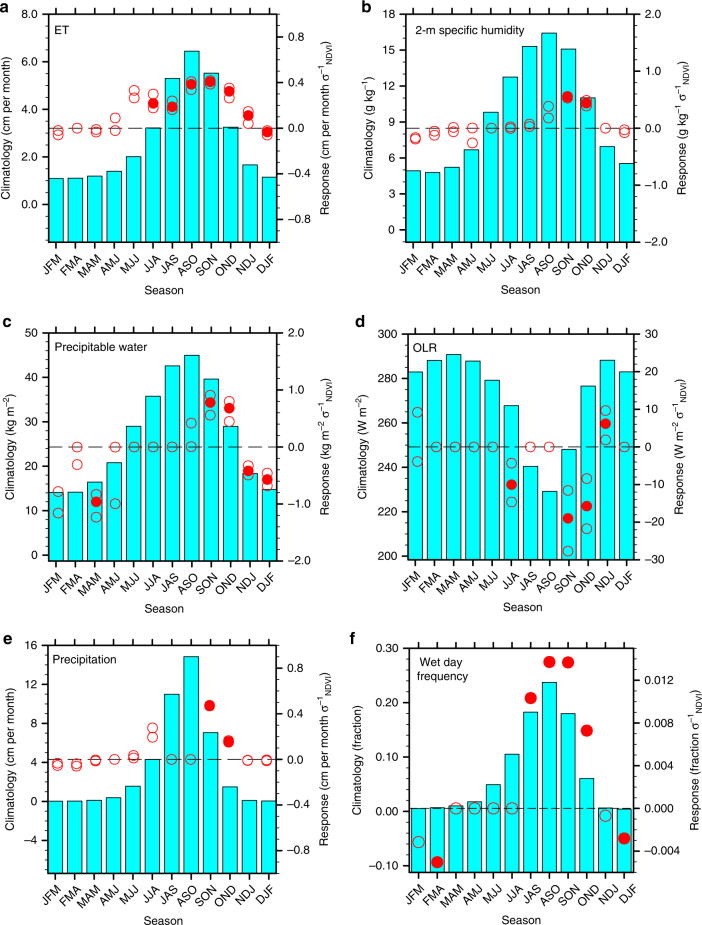
Multi-data set observational moisture responses to positive local NDVI anomalies across the Sahel during 1982–2011. **a** evapotranspiration (ET), **b** 2-m specific humidity, **c** precipitable water, **d** outgoing longwave radiation (OLR), **e** precipitation, and **f** frequency of wet days. In **a**–**e**, dots indicate statistically significant (*p* < 0.1) multi-data set average responses, referring to the right y-axis; open circles represent the 10th and 90th percentiles of the multi-data set responses, regardless of their statistical significance. In **f**, dots and open circles indicate significant and insignificant responses, respectively, using daily station precipitation data. Dashed lines indicate a response of zero. Bars indicate the multi-data set mean climatology of the response variable, referring to the left y-axis. σ_NDVI_ in the units of the response variables refers to one standard deviation in the Sahel NDVI anomaly. Labels on the x-axis stand for 3-month seasons, e.g., JFM for January, February, and March. The statistical significance is determined based on Monte Carlo bootstrap testing

The relatively enhanced contribution from land-atmosphere interactions in autumn is likely attributed to two factors. One likely explanation is the reduced amplitude and broad-scale atmospheric circulation impacts of key ocean-atmosphere teleconnection patterns^33^, including El Niño-Southern Oscillation (ENSO)^34–36^ and Atlantic Niño mode^37^ (Supplementary Fig. 3). Another contributing factor involves seasonally wet soils^38^ and consequential vegetation growth in response to the antecedent monsoon that support significant evapotranspiration (ET) fluxes (Fig. 2a). Indeed, the explained variance in Sahel ET associated with terrestrial drivers peaks during SON at 51%, according to the multi-data set average. Note that the apparent greater contribution from oceanic forcings is not an artifact of the imbalanced number of oceanic versus terrestrial forcings, as confirmed by additional sensitivity tests that modulate the number of either type of forcing.

Similar to the prior observational study based on a statistical vegetation index^23^, the combined oceanic and terrestrial drivers considered here through GEFA explain only 20–52% and 35–75%, by month, of the total variance in observed Sahel precipitation and air temperature, respectively. The residual portion of the total variance in precipitation and air temperature is comprised of the atmospheric internal variability (equation 1 in Methods section), non-linear impacts of oceanic and terrestrial forcings which are not detected by the linear GEFA statistical method, and impacts from other oceanic (e.g., higher order SST empirical orthogonal functions (EOFs) or SST EOFs from other basins) or terrestrial (e.g., NDVI from other ecoregions) forcings absent from the forcing matrix. Measurement errors, which have been partly accounted for in the analysis, potentially contribute to this residual explained variance as well. In contrast to the previous observational study based on Granger causality analysis^25^, the current GEFA-based analysis includes both nearby and remote oceanic forcings and considers multiple observational datasets, thereby providing a more convincing quantification of oceanic versus terrestrial contributions to Sahel rainfall variability. Furthermore, by considering both nearby and remote oceanic impacts, the GEFA-based assessment of oceanic versus terrestrial contributions is directly comparable with previous modeling studies that modify global SSTs through dynamical experiments^10, 12^.

### Vegetation-climate feedbacks in the Sahel

The observational analysis verifies the proposed positive vegetation-rainfall feedback across the Sahel from prior modeling studies^6, 10, 12, 13^ and regression-based observational studies^23–26^. However, the feedback is seemingly confined to the post-monsoon autumn season and largely due to a moisture recycling mechanism (Fig. 2). Positive NDVI anomalies favor enhanced ET, precipitable water, convective activity [reduced outgoing longwave radiation (OLR)], and total precipitation amount, indicative of amplified moisture recycling. The positive vegetation-rainfall feedback in the Sahel is confirmed with station rainfall observations (Supplementary Fig. 4a) and regional downscaling of global reanalysis (Supplementary Note 1, Supplementary Fig. 5a). The ET and precipitation responses are of comparable magnitude in SON, namely +0.43 (0.40–0.44 among data sets) cm month^−1^ per standard deviation of NDVI anomalies (σ^−1^_NDVI_) and +0.49 (0.48–0.50 among data sets) cm month^−1^ σ^−1^_NDVI_, respectively, implying the dominance of the moisture recycling mechanism underlying the positive vegetation-rainfall feedback in the post-monsoon season across the Sahel (Fig. 2a, e). Vegetation imposes a greater influence on the frequency, rather than intensity, of convective activity across the Sahel, consistent with previous observational findings regarding enhanced probability of afternoon precipitation in eastern United States and Mexico by high evaporation^39^. Specifically, the increase in precipitation amount of +7.7 (7.5–7.9 among data sets) % σ^−1^_NDVI_ during SON is largely due to a +7.8% σ^−1^_NDVI_ increase in precipitation frequency, with minimal response in precipitation event intensity to NDVI anomalies (Fig. 2e, f). A likely explanation for the differential response in precipitation frequency versus intensity is that the surface turbulent flux partitioning, associated with the vegetation and soil moisture anomalies, shifts the local atmosphere from a non-convective to convective state, while other broad-scale controls, such as free tropospheric moisture content or large-scale moisture convergence, largely determine the rainfall intensity^40^. Furthermore, positive NDVI anomalies favor a higher frequency of moderately low OLR days in the Sahel (not shown), implying that anomalously enhanced vegetation growth supports an increased chance of moderate-intensity convective events. Although the GEFA-based analysis agrees with previous regression-based analyses on the existence of a positive vegetation-rainfall feedback in the Sahel, the statistical vegetation index analysis concluded that the vegetation feedback peaks during the monsoon season^23^, rather than the post-monsoon season as identified by GEFA. A potential reason for this inconsistent conclusion is that the regression-based analysis failed to account for the oceanic impacts, which peak during the monsoon season (Fig. 1) and likely bias the estimated vegetation impacts. The identified positive vegetation-rainfall feedback in the Sahel during the late-to-post monsoon season suggests that an anomalous wet monsoon tends to persist longer, since enhanced vegetation growth and wet soil associated with anomalously abundant rainfall likely cause an extended monsoon season over the Sahel. This proposed link between an anomalously wet and extended monsoon season is supported by a temporal correlation of 0.58 between the observed detrended JJA rainfall and September rainfall anomalies over the Sahel during 1901–2014.

**Fig. 3:**
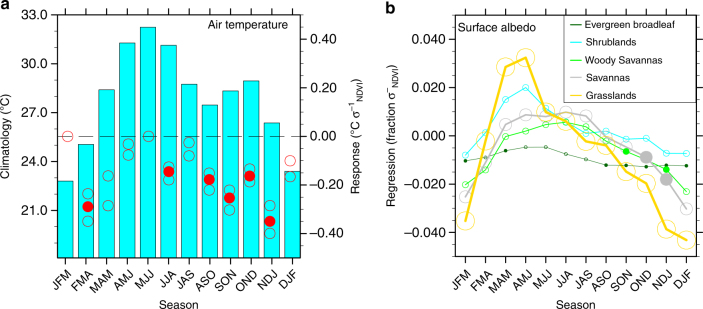
Observed local energy responses to Sahel NDVI anomalies during 1982–2011. **a** surface air temperature and **b** temporal regression coefficient of monthly surface albedo upon standardized NDVI anomalies, averaged by biome type. In **a**, dots indicate statistically significant (*p* < 0.1, based on Monte Carlo bootstrap test) multi-data set average responses, referring to the right *y* axis; open circles represent the 10th and 90th percentiles of the multi-data set responses, regardless of their statistical significance; the dashed line indicates a response of zero; bars indicate the multi-data set mean climatology of the response variable, referring to the left *y* axis. In **b**, filled dots indicate statistically significant (*p* < 0.1) correlations, according to the Student’s t-test; the size of the circles and thickness of lines denote the annual abundance of the corresponding biome across the Sahel, which is based on the remotely sensed land cover type from the International Satellite Land Surface Climatology Project (ISLSCP) initiative II International Geosphere-Biosphere Project (IGBP) DISCover and Simple Biosphere (SiB) Land Cover data set. The surface albedo is from the Global Land Surface Satellites data set^66^. In both **a** and **b**, labels on the *x* axis stand for 3-month seasons, e.g., JFM for January, February, and March

The observational analysis here, focused on the seasonal to interannual time scale, challenges the proposed classic albedo-based mechanism^6^ for Sahel vegetation feedbacks. Low-level cooling (due to increased ET and latent heat flux and thus decreased Bowen ratio), weakened lapse rate (Fig. 3a), higher surface pressure, and anomalous lower-tropospheric to mid-tropospheric subsidence (Supplementary Fig. 6) in response to positive NDVI anomalies support an active stability mechanism, consistent with findings in the regional modeling study regarding the impacts of vegetation cover on heat and moisture fluxes over the Sahel and West African monsoon region^41^. The increased atmospheric stability in response to positive NDVI anomalies, although swamped by the moisture recycling mechanism, is counter to the dynamic responses expected through the classic vegetation-albedo feedback mechanism. During autumn, positive NDVI anomalies trigger modest declines in surface albedo, confined to the savanna, woody savanna, and broadleaf evergreen forest portions of the Sahel (Fig. 3b), consistent with previous model-based findings^42^. However, the magnitudes of the albedo anomalies, on the order of 0.01–0.02 σ^−1^_NDVI_, are too small, compared with the imposed change in surface albedo of 0.21 applied in the pioneering experiments of Charney^6^, to trigger the instability responses necessary for the albedo-based positive vegetation-rainfall feedback. The albedo responses are trivial over the more widespread grasslands or shrublands of the Sahel, probably because the seasonal to interannual time scale is too short for the grass-desert or shrub-desert conversions proposed by Charney^6^. There is observed evidence of a weak vegetation roughness mechanism^43^, with slightly diminished 10-m wind speed on the order of −0.05 (−0.04 to −0.08 among data sets) m s^−1^ σ^−1^_NDVI_ during July–September, in response to positive NDVI anomalies (Supplementary Fig. 6a). The lack of anomalous ascending motion in response to enhanced vegetation abundance (Supplementary Fig. 6c), however, suggests that both the albedo and roughness mechanisms are swamped by the dominant moisture recycling feedback mechanism in the Sahel on seasonal to interannual time scales. However, at long time scales associated with land use change, the albedo impact might be significantly more important if pronounced grass–desert conversion or soil degradation occurs. The surface cooling and weakened surface wind speed associated with positive NDVI anomalies are confirmed with station observations (Supplementary Fig. 4b, c) and regional downscaling of global reanalysis (Supplementary Note 1, Supplementary Fig. 5b–d) in terms of both sign and magnitude.

**Fig. 4:**
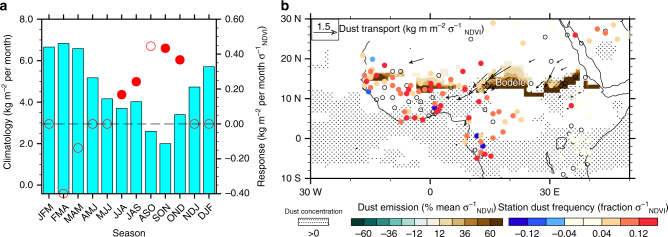
Observed dust responses to negative Sahel NDVI anomalies and corresponding dry soil anomalies during 1982–2011. **a** total dust emission (bars: climatology, referring to the left *y* axis; dots and open circles: significant (*p* < 0.1 based on Monte Carlo bootstrap test) and insignificant responses, respectively, referring to the right *y* axis). In **a**, labels on the *x* axis stand for 3-month seasons, e.g., JFM for January, February, and March. **b** spatial pattern of GEFA responses in dust emission (% climatology σ_NDVI_^−1^, green-brown color), column dust concentration (kg m^−2^ σ_NDVI_^−1^, stitching and hatching), column dust transport (kg m^−1^ per month σ_NDVI_^−1^, vector), and regional dust frequency (defined in Methods section, fraction σ_NDVI_^−1^, circles in blue-red color) in September-November. Only statistical significant (*p* < 0.1) responses are shown in **b**, according to the Monte Carlo bootstrap test. Open circles in **b** represent stations with insignificant responses in dust frequency

The observational GEFA analysis further verifies that diminished vegetation growth and accompanying dry soils across the Sahel lead to enhanced dust emissions and dust storm activity during the mid- to post-monsoon season (Fig. 4a), as suggested by previous correlation-based observational studies^44^. In addition, the current observational analysis reveals the remote impacts of Sahel vegetation and soil moisture on dust concentration over the tropical Atlantic Ocean. The enhancement in dust emissions is most pronounced across the southern boundary of North Africa’s major dust source regions within the Sahel, including the Bodélé Depression^45^, where dust emissions increase by more than 60% during SON in response to a negative NDVI anomaly on the order of one standard deviation (Fig. 4b). These enhanced emissions across the Sahel support increased southwestward dust transport and thus elevated surface and column dust concentrations, as well as greater frequency of dust days according to station observations (defined in Methods section), across tropical and subtropical North Africa and the eastern tropical Atlantic Ocean (Fig. 4b). This observed vegetation-dust feedback acts as a potential secondary mechanism for the positive vegetation-rainfall feedback in the Sahel, as proposed by previous modeling studies^46, 47^, given the direct effects of dust aerosols that cause low-level cooling and atmospheric stabilization, and the indirect radiative effects of dust aerosols that increase the number of cloud condensation nuclei and inhibit precipitation efficiency.

The current study presents the first convincing observational evidence for the model-hypothesized positive vegetation-rainfall feedbacks in the Sahel, by successfully isolating terrestrial feedbacks from oceanic drivers and systematically examining multiple observational datasets in order to quantify observational uncertainty in feedback response estimates. The identification of key oceanic and terrestrial drivers will aid in successful seasonal predictions of regional climate in Sahel, a region that is highly vulnerable to hydrological extremes.

Future projections of Sahel rainfall, in response to the anthropogenically enhanced greenhouse effect, remain highly uncertain in terms of both sign and magnitude within phases three and five of the Coupled Model Intercomparison Project (CMIP3 and CMIP5)^1, 14, 15, 48, 49^. Indeed, the Sahel is one of the most uncertain regions for rainfall projections worldwide. In further studies, the GEFA-based assessment of the key observed oceanic and terrestrial drivers of North African regional climate will serve as an observational benchmark for evaluating the representation of ocean-land-atmosphere interactions within state-of-the-art climate models as applied by the Intergovernmental Panel on Climate Change. This innovative approach will foster model evaluation and development, along with the formulation of process-based model performance metrics for weighting future climate projections for the Sahel and reducing associated uncertainty.

## Methods

### GEFA and stepwise selection

In the current study, a multivariate statistical method, GEFA, is used to study the oceanic and terrestrial regulators of the Sahel’s climate. The statistical GEFA approach extracts the forcing of a slowly-evolving environmental variable, such as SST or NDVI, on the rapidly-evolving atmosphere, either in climate model output or observational data. The GEFA methodology^29^, based on stochastic climate theory^50^, addresses the local and non-local feedbacks simultaneously, which is critical given that vegetation and SST anomalies can affect atmospheric conditions both locally and remotely^19, 50, 51^. GEFA can largely separate the individual impacts of different ocean basins and vegetated regions on climate in select regions. For example, GEFA’s capability of extracting the oceanic and terrestrial impacts on North American regional climate was previously demonstrated using the NCAR Community Climate System Model Version 3.5^19, 30^. In particular, GEFA’s reliability in isolating the oceanic and land surface feedbacks on the North African climate was successfully demonstrated by comparing the statistical assessments with dynamical experiments in CESM^31, 32^. In the GEFA validation regarding the assessed terrestrial impacts on North African climate, the statistically assessed atmospheric responses were evaluated against those assessed from two ensembles of dynamical experiments developed for the Sahel or West African Monsoon (WAM) region, i.e., EXP_LAI_, in which regional leaf area index (LAI) was modified, and EXP_SOIL_, in which regional LAI and soil moisture were modified together during winter-spring, motivated by the strong soil moisture-LAI coupling across the Sahel and the WAM region in CESM.

At time scales longer than the atmospheric memory (about 1 week), the atmospheric variable (e.g. precipitation) at time *t*, *A(t)*, can be expressed as the sum of feedback responses to an array of slowly-evolving variables (e.g. SST, NDVI), **O**(*t*), and the atmospheric internal noise, *N(t)* ^29^:

1 $$A\left( t \right) = {\mathbf{B}} \cdot {\mathbf{O}}\left( t \right) + N(t),$$

where **B** is the feedback matrix. Right multiplying **O** ^*T*^(*t-τ*) on both sides of equation (1) and applying the covariance yield:

2 $${\mathbf{C}}_{A{\mathbf{O}}}\left( \tau \right) = {\mathbf{B}} \cdot {\mathbf{C}}_{{\mathbf{OO}}}\left( \tau \right) + {\mathbf{C}}_{N{\mathbf{O}}}(\tau ),$$

where *τ* is the time scale, exceeding the atmospheric adjustment time, and **C**(*τ*) represents a covariance matrix at lag *τ*. Given the time series’ length *L* of the atmospheric and oceanic variables, the lagged covariance matrices are estimated as:

3 $${\mathbf{C}}_{A{\mathbf{O}}}\left( \tau \right) = \frac{1}{L}A\left( t \right){\mathbf{O}}^T\left( {t - \tau } \right),{\mathbf{C}}_{{\mathbf{OO}}}\left( \tau \right) = \frac{1}{L}{\mathbf{O}}\left( t \right){\mathbf{O}}^T\left( {t - \tau } \right),{\mathbf{C}}_{N{\mathbf{O}}}\left( \tau \right) = \frac{1}{L}N\left( t \right){\mathbf{O}}^T\left( {t - \tau } \right).$$

The superscript *T* indicates a transposed matrix. Since oceanic or terrestrial variability cannot be forced by an atmospheric internal noise at a later time, and the atmospheric internal noise is not affected by oceanic or terrestrial variability by definition in equation (1), **C**_*N***O**_(*τ*) = 0, which results in an estimate of the feedback matrix as:

4 $${\mathbf{B}} = {\mathbf{C}}_{A{\mathbf{O}}}(\tau ) \cdot {\mathbf{C}}_{{\mathbf{OO}}}^{ - 1}(\tau ).$$

The estimated feedback matrix represents the instantaneous influence of slowly-evolving oceanic and terrestrial variables on an atmospheric variable. In theory, the estimate of **B** does not depend on *τ*, but due to insufficient *L*, the sampling error always increases with greater *τ*, resulting in unrealistic estimates of the magnitude of the feedback response at large *τ*. On the basis of the GEFA evaluation work within CESM^31, 32^, larger *τ* leads to deteriorating magnitude estimates of the oceanic and terrestrial feedbacks compared to the dynamical experiments, especially with shorter data records that are comparable in length with most observational data sets. Therefore, *τ* is assigned to be one month in the current observational analysis.

In the current study, the GEFA forcing matrix is comprised of the leading two SST EOF modes from eight non-overlapping basins, area-average Mediterranean SSTs (Supplementary Fig. 7), and time series of area-average NDVI across the Sahel (12˚ N–17˚ N, 20˚ W–40˚ E), WAM region (5˚ N–12˚ N, 20˚ W–30˚ E), and Horn of Africa (HOA) (5˚ S–10˚ N, 30˚ E–52˚ E) (Supplementary Fig. 8). Higher order SST EOF modes are less important, as evidenced by the trivial increase in total explained variance by adding the third to fifth EOF modes of basin SSTs to the forcing matrix. The purpose of performing GEFA in truncated SST EOF space is to reduce the sampling error from highly correlated forcing fields^51, 52^. Past studies have suggested the potential impacts of SST variability across a vast multitude of ocean basins, including the tropical Pacific (TP)^3, 53^, North Pacific (NP), tropical Atlantic (TA)^5, 53–55^, tropical Indian (TI)^53, 56^, North Atlantic (NA)^1, 54^, South Pacific (SP)^3^, South Indian (SI)^3^, South Atlantic (SA)^3^, and Mediterranean Sea^57^, on Sahel rainfall. Moreover, for most basins, the leading two EOF modes have clear physical meanings, such as ENSO, Indian Ocean Basin Mode, and Atlantic Niño mode. In terms of terrestrial forcings, the Sahel, WAM region, and HOA represent unique North African landscapes, i.e. mainly savanna and grasslands across the Sahel, savanna and woody savanna across the WAM region, and shrubs and bare ground across the HOA. In order to obtain reliable estimates of vegetation feedbacks in the Sahel, land forcings that are moderately-to-highly correlated with NDVI in the Sahel, such as NDVI in the WAM and HOA, also need to be included in the forcing matrix^32^. The geographic extent of the three ecoregions is determined through rotated EOF analysis of detrended monthly remotely sensed NDVI anomalies. Regional average responses are obtained by applying GEFA to the atmospheric fields averaged across the Sahel (12˚ N–17˚ N, 20˚ W–40˚ E). The analysis is performed for 1982–2011. Before applying GEFA, the seasonal cycle and linear trend are removed from all forcing and response fields. The statistical significance of GEFA feedback matrices is assessed using the Monte Carlo bootstrap method with 1000 random iterations in which the time series of the response variable is scrambled^30^. In order to achieve sufficient length of data and obtain reliable estimates of the feedback matrices (explained later), seasonal feedbacks are estimated by aggregating data from the consecutive three months so that the effective sample size is three times the number of years, or 90 months.

The short duration of the remotely sensed NDVI record, covering about three decades, remains a challenge to the application of GEFA to understand land-atmosphere feedbacks, especially when simultaneously considering numerous potential forcings. In order to obtain reliable seasonal estimates of the feedback matrix (**B**) in terms of about 20 forcings (17 SST fields and 3 NDVI fields) for the Sahel, at least 100 years of data is needed for most response variables so that GEFA can accurately capture the seasonal cycle of area-average seasonal responses with a temporal correlation of 0.8 or greater (*N* = 12 months) with the dynamical experiments, which are regarded as the truth and provide a benchmark for GEFA evaluation^32^. In order to minimize the sampling error associated with relatively short datasets, it is necessary to reduce the number of forcings under consideration before estimating the feedback matrix. Here negligible forcings for the Sahel are eliminated using a backward-selection stepwise method^58^, which compares the relative contribution from each forcing to the atmospheric variability and retains the truly important ones as predictors through an automated procedure. Stepwise selection has been widely applied to predictor selection in developing linear prediction models for the climate or ecosystems^59, 60^. Akaike information criterion (AIC)^61^, which measures the relative quality of a statistical model by estimating the goodness of fit and penalizing the complexity of the model (number of predictors), is used as the criterion in the stepwise selection.

5 $${\rm AIC} = 2 \times {N_f} - 2 \times \ln \left( {\hat L} \right),$$

6 $$\hat L = - \frac{L}{2}\ln \left(\mathop {\sum }\limits_{t = 1}^L \left( {\hat A\left( t \right) - A\left( t \right)} \right)^2/L\right) + C_1,$$

7 $$\hat A(t) = {\mathbf{B}} \cdot {\mathbf{O}}(t).$$

In equation (5), *N*_*f*_ represents the number of forcings in the forcing matrix, and $\hat L$ stands for the maximized likelihood function of the statistical model in (1), which represents the goodness of fit of the statistical model in (1) with the **B** matrix obtained from equation (4) and is estimated by equations (6) and (7), based on the linear theory^62^. In equation (6), *L* is the length of the data record, and *C*_1_ is a constant independent of the statistical model. In equation (7), $\hat A(t)$ stands for the predicted atmospheric condition at time *t*, based on equation (1). If AIC does not increase after removing a select forcing, then this forcing has negligible contribution to the variability of the atmospheric variable and can subsequently remain excluded from the forcing matrix. In this way, the number of forcings to be assessed by GEFA decreases, thereby allowing more reliable estimates of the feedbacks associated with the remaining, significant forcings. The length of data needed to obtain reliable seasonal estimates of the feedback matrix (**B**), such that GEFA can capture the seasonal cycle of area-average responses for most variables, including ET, surface air temperature, planetary boundary layer height, surface specific humidity, surface wind, and rainfall^32^, with a temporal correlation of at least 0.8 (*N* = 12 months) with the dynamical experiments, is reduced to about 30 years by applying this stepwise selection procedure^32^.

According to GEFA, the percent variance in a select response variable, as explained by either an oceanic or terrestrial forcing, is calculated similarly to the analysis of variance (ANOVA) approach in multiple linear regression^62^. For example, the percentage of explained variance by oceanic forcings, *V*_**O**_, is calculated by

8 $$V_{\mathbf{O}} = C_{{\mathbf{A}}_{\mathbf{O}}{\mathbf{A}}}/V_{\mathbf{A}},$$

where $C_{{\mathbf{A}}_{\mathbf{O}}{\mathbf{A}}}$ is the covariance between the observed atmospheric time series (**A**) and predicted atmospheric time series by oceanic forcings (**A**_**O**_), and *V*_**A**_ is the variance of the atmospheric time series. The predicted atmospheric time series is reconstructed by

9 $${\mathbf{A}}_{\mathbf{O}} = {\mathbf{B}}_{\mathbf{O}} \cdot {\mathbf{O}},$$

where **B**_**O**_ is the GEFA feedback matrix when only oceanic forcings are included in the forcing matrix, and **O** is the forcing matrix containing the oceanic forcings. The percentage of explained variance by terrestrial forcings is calculated similarly.

### Multi-data set bootstrapping method

Multiple observational, remote sensing, and reanalysis datasets (Supplementary Table 1) are analyzed for the Sahel in the GEFA framework. By applying the Monte Carlo bootstrapping approach^63^, the potential impacts of observational measurement errors across data-limited North Africa on estimated GEFA response fields are reduced. Furthermore, this approach facilitates a reliable estimation of the multi-data set mean and quantification of observational uncertainty in the GEFA-based atmospheric responses to oceanic and terrestrial forcings. The GEFA-based response is first obtained from each data set, and then a probability distribution function (PDF) of the weighted-average response of all datasets is generated by the Monte Carlo bootstrap approach with 1000 random iterations. With each iteration, weights are randomly generated from a uniform distribution and assigned to the datasets in the order of their regional reliability, with the highest weight assigned to the most reliable data set. On the basis of the multi-data set PDF of the 1000 weighted averages, the multi-data set average and uncertainty range of the responses to oceanic and terrestrial forcings are obtained. The regional reliability of each observational data set across the Sahel is evaluated based on the criteria outlined in Supplementary Tables 2–6, leading to a practical ranking of all data sets, specifically for the Sahel, to be applied in the Monte Carlo bootstrap approach^7^.

### Station dust observations and MERRA-2 dust reanalysis

Dust observations are retrieved from the National Climatic Data Center (NCDC) hourly global and U.S. Integrated Surface hourly data set for 1982–2011 at 502 stations across North Africa. At each station, a dust day is defined as a day in which either dust/sand storm or severe dust/sand storm is reported at least once, or dust suspension is reported for at least a quarter of the total number of observations during the daytime^60, 64^. Therefore, the dust day metric is a combined measure of the frequency and intensity of dust activity.

Station observations are first gridded to a spatial resolution of 0.25˚ × 0.25˚. In each grid cell, a regional dust day is defined if at least one station within that grid cell indicates a dust day. Monthly dust frequency in each grid cell is calculated when dust observations are available on more than half of the days during that month, or otherwise left as a missing value.

In addition to station dust observations, dust aerosol reanalysis from MERRA-2 is also analyzed. The MERRA-2 reanalysis of aerosols includes assimilation of bias corrected Aerosol Optical Depth (AOD) from Advanced Very High Resolution Radiometer (AVHRR) over ocean, Moderate Resolution Imaging Spectroradiometer (MODIS) sensors on both Terra and Aqua satellites, Multi-angle Imaging SpectroRadiometer (MISR) over bright surfaces and Aerosol Robotic Network (AERONET) data. The vertical structure of MERRA-2 aerosol reanalysis has been successfully validated using Cloud-Aerosol Lidar with Orthogonal Polarization (CALIOP) data over a number of regions, including North Africa during 2008^65^. Furthermore, the MERRA-2 surface dust concentration reanalysis exhibits similar seasonal cycle and interannual variability with the station dust frequency across the Sahel (not shown).

### Data availability

The data that support the findings of this study are available from the corresponding authors upon request.

## Electronic supplementary material

Peer Review File

Supplementary Information
