# Supplementary material for: Observed positive vegetation-rainfall feedbacks in the Sahel dominated by a moisture recycling mechanism
Source: Nat Commun. 2017 Nov 30;8:1873. doi: 10.1038/s41467-017-02021-1 (PMC5707399; doi:10.1038/s41467-017-02021-1)
Supplement: Supplementary file 2 — Supplementary Information [file 41467_2017_2021_MOESM2_ESM.pdf]

Supplementary Note 1 | CORDEX-Africa downscaled regional climate data

Recent decline in the number of stations across North Africa causes uncertainty in the gridded precipitation and air temperature datasets[1]. In addition, the coarse spatial resolution in global reanalysis data has potential influence on the assessed terrestrial feedbacks in the Sahel, which exhibits strong climatic and ecological gradients. In light of these uncertainties in the observational and reanalysis data, downscaled regional climate data from the Coordinated Regional Climate Downscaling Experiment over the Africa domain (CORDEX-Africa)[2] is also examined. The analysis focuses on output from eight regional climate models based on ERA-Interim, covering more than 20 years during 1979-2012 with a spatial resolution of 0.44˚ latitude x 0.44˚ longitude. The assessed responses in precipitation, surface air temperature, surface wind speed, and sea-level pressure to NDVI anomalies in the Sahel are largely consistent with those presented in the main text using gridded observations and global reanalyses (Supplementary Figure 5).

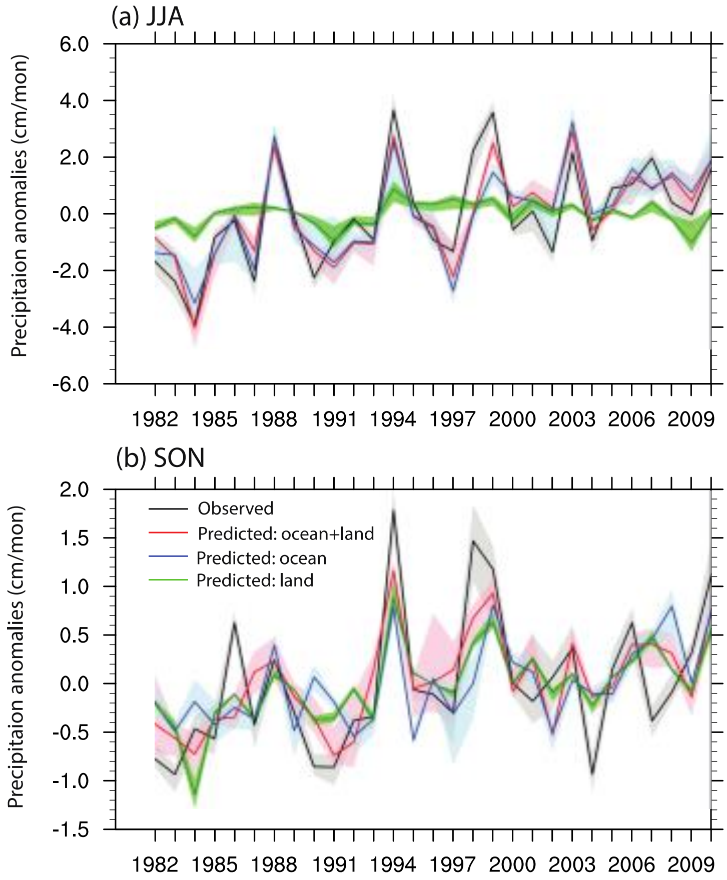

Supplementary Figure 1 | Sahel precipitation in (a) JJA and (b) SON during 1982-2010 from observations (black line with grey shading), and predicted based on GEFA using all oceanic and terrestrial forcings (red line with pink shading), only oceanic forcings (blue line with blue shading), and only terrestrial forcings (green line with green shading). The predicted time series are obtained through the leave-one-out approach, in which the GEFA feedback matrix is estimated using all years' data except the targeting year. The temporal correlations between the predicted and observed SON (JJA) precipitation are 0.81 (0.88), 0.56 (0.84), and 0.79 (0.43) (all p's < 0.1) using both oceanic and land forcings, only oceanic forcings, and only land forcings, respectively. Solid lines and shading represent the multi-dataset average and the multi-dataset minimum and maximum, respectively.

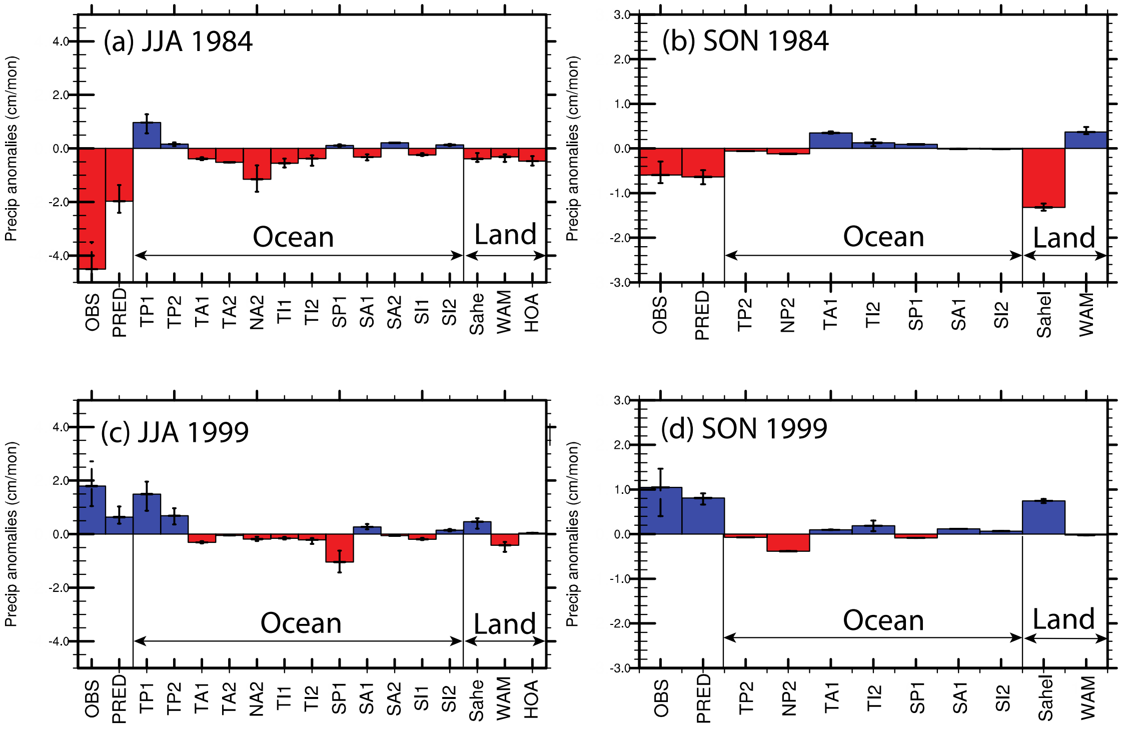

Supplementary Figure 2 | Decomposing the contribution (cm month^{-1}) from each oceanic and terrestrial forcing on the Sahel's precipitation anomalies in 1984 (a and b) and 1999 (c and d) during the monsoon (JJA, a and c) and post-monsoon (SON, b and d) seasons, based on GEFA. The contribution from each individual forcing is calculated as the product of the feedback coefficient and forcing anomaly[3]. Only forcings selected by stepwise GEFA are presented. Bars (error bars) represent the multi-dataset average (10th and 90th percentiles) contribution. The two leftmost bars represent the observed and predicted precipitation.

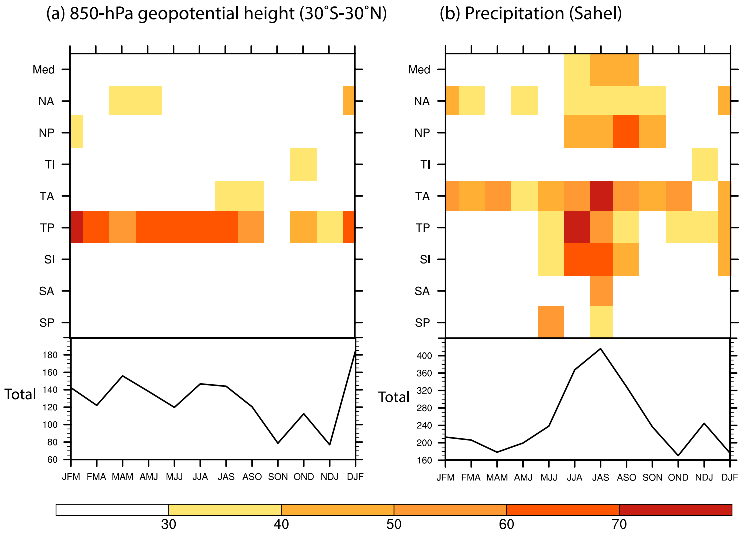

Supplementary Figure 3 | Strength of oceanic regulation of atmospheric conditions by season. Multi-dataset average percent area of significant ($p<0.1$) observed responses to at least one of two leading SST EOFs in each oceanic basin in (a) 850-hPa geopotential height across the entire Tropics, specifically 30˚S-30˚N, and (b) precipitation across the Sahel. The lines represent the sum of the percent area of significant ($p<0.1$) observed responses to all oceanic basins. Labels on the x-axis stand for three-month seasons, e.g. JFM for January, February and March.

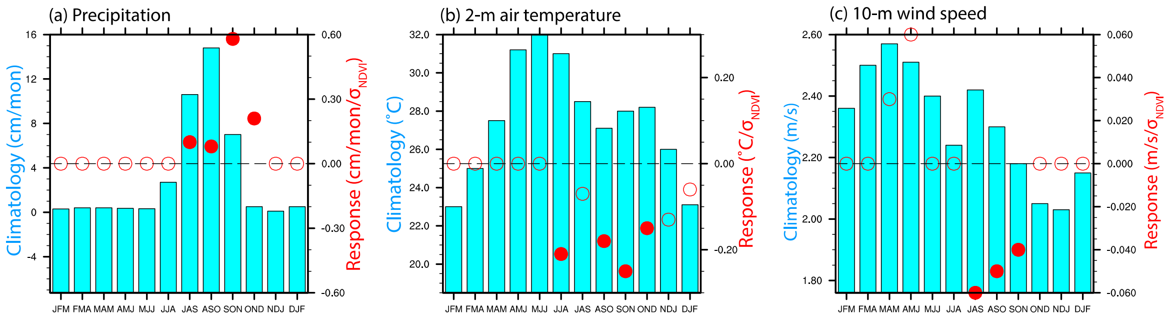

Supplementary Figure 4 | Observed response to positive local NDVI anomalies across the Sahel based on 87 weather stations during 1982-2011: (a) precipitation, (b) 2-m air temperature, and (c) 10-m wind speed. Dots and open circles indicate significant and insignificant responses, respectively, referring to the right y-axis. Bars indicate the climatology of the response variable, referring to the left y-axis. Labels on the x-axis stand for three-month seasons, e.g. JFM for January, February and March.

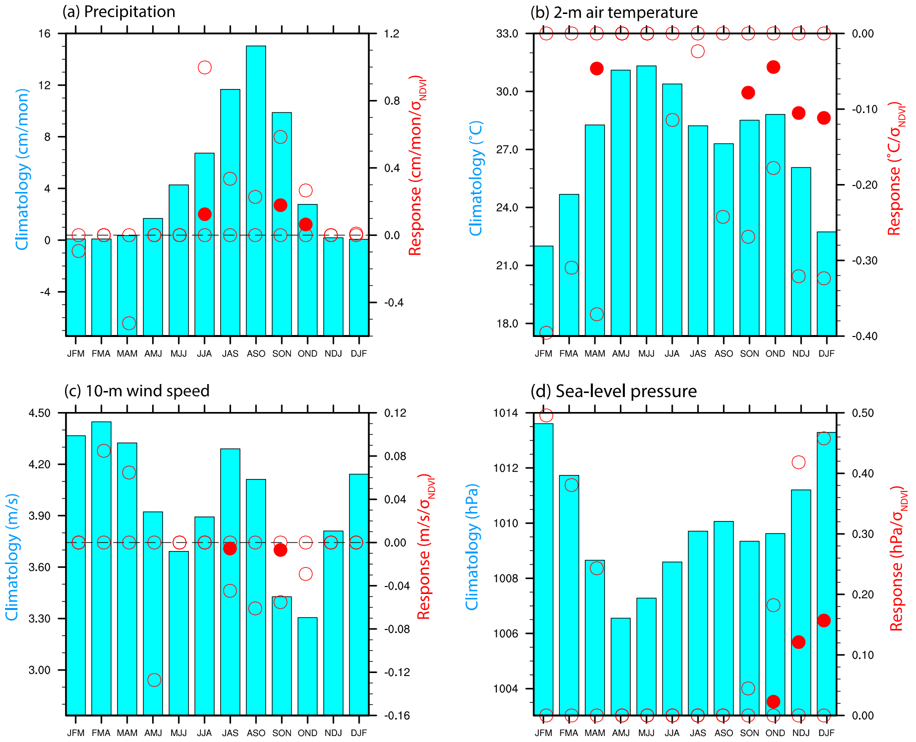

Supplementary Figure 5 | Multi-data response to positive local NDVI anomalies across the Sahel from CORDEX-Africa[2] multi-model downscaled regional climate data based on ERA-Interim. (a) Precipitation, (b) 2-m air temperature, (c) 10-m wind speed, and (d) sea-level pressure. Dots indicate statistically significant ($p<0.1$) multi-data average responses, referring to the right y-axis; open circles represent the minimum and maximum of the multi-data responses, regardless of their statistical significance. Bars indicate the multi-data mean climatology of the response variable, referring to the left y-axis. Labels on the x-axis stand for three-month seasons, e.g. JFM for January, February and March.

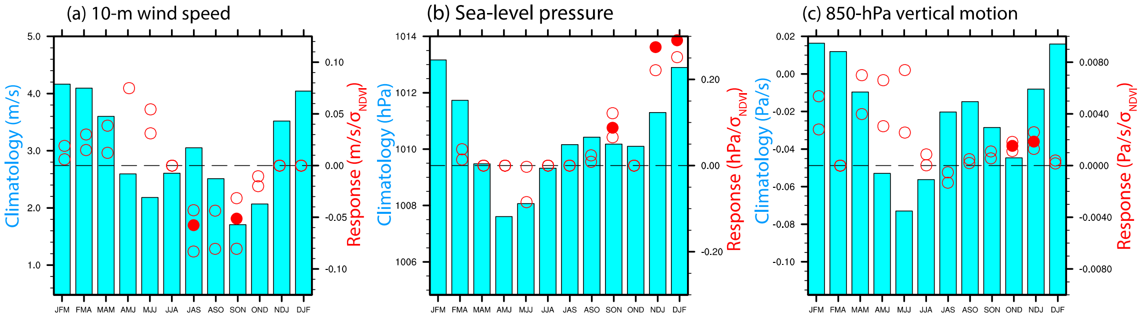

Supplementary Figure 6 | Multi-data observed momentum response to positive local NDVI anomalies across the Sahel. (a) 10-m wind speed, (b) sea-level pressure, and (c) 850-hPa vertical motion (positive for anomalous subsidence). Dots indicate statistically significant ($p<0.1$) multi-dataset average responses, referring to the right y-axis; open circles represent the 10th and 90th percentiles of the multi-dataset responses, regardless of their statistical significance. Bars indicate the multi-dataset mean climatology of the response variable, referring to the left y-axis. Labels on the x-axis stand for three-month seasons, e.g. JFM for January, February and March.

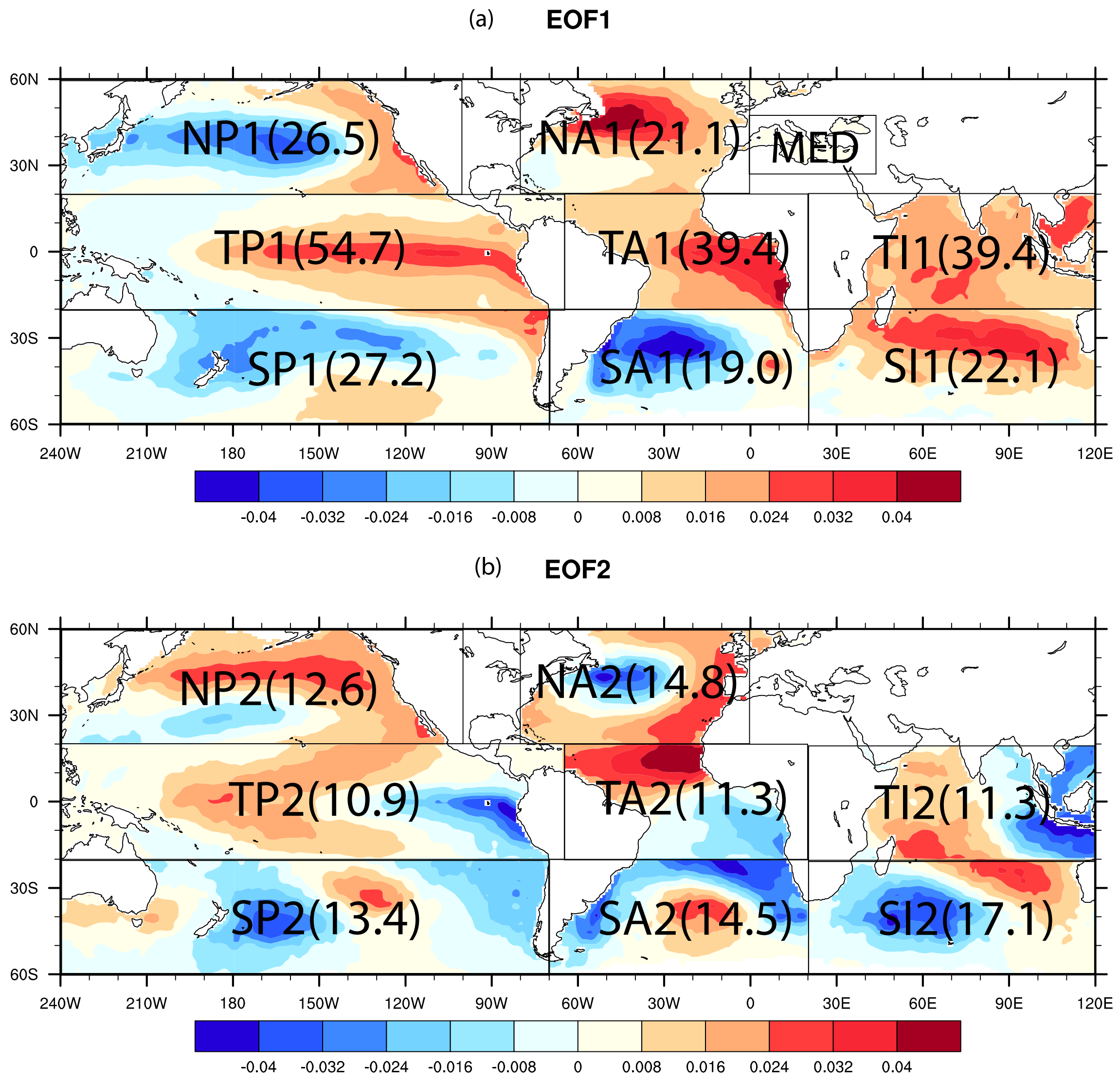

Supplementary Figure 7 | Collage of the spatial patterns of the observed (a) first and (b) second EOF modes (unitless) of SST anomalies in eight ocean basins. The oceanic basins include the tropical Pacific (TP), North Pacific (NP), tropical Indian (TI), tropical Atlantic (TA), North Atlantic (NA), South Pacific (SP), South Indian (SI), and South Atlantic (SA). The area-average Mediterranean [MED in (a)] SST is also included in the forcing matrix. Analysis is based on the Met Office – Hadley Centre Global Sea Ice Coverage and SST dataset for 1900-2011[4]. The EOF analysis is performed within each individual basin separately. The percentage of explained variance in SST anomalies across a specific oceanic basin by each mode is identified in parentheses.

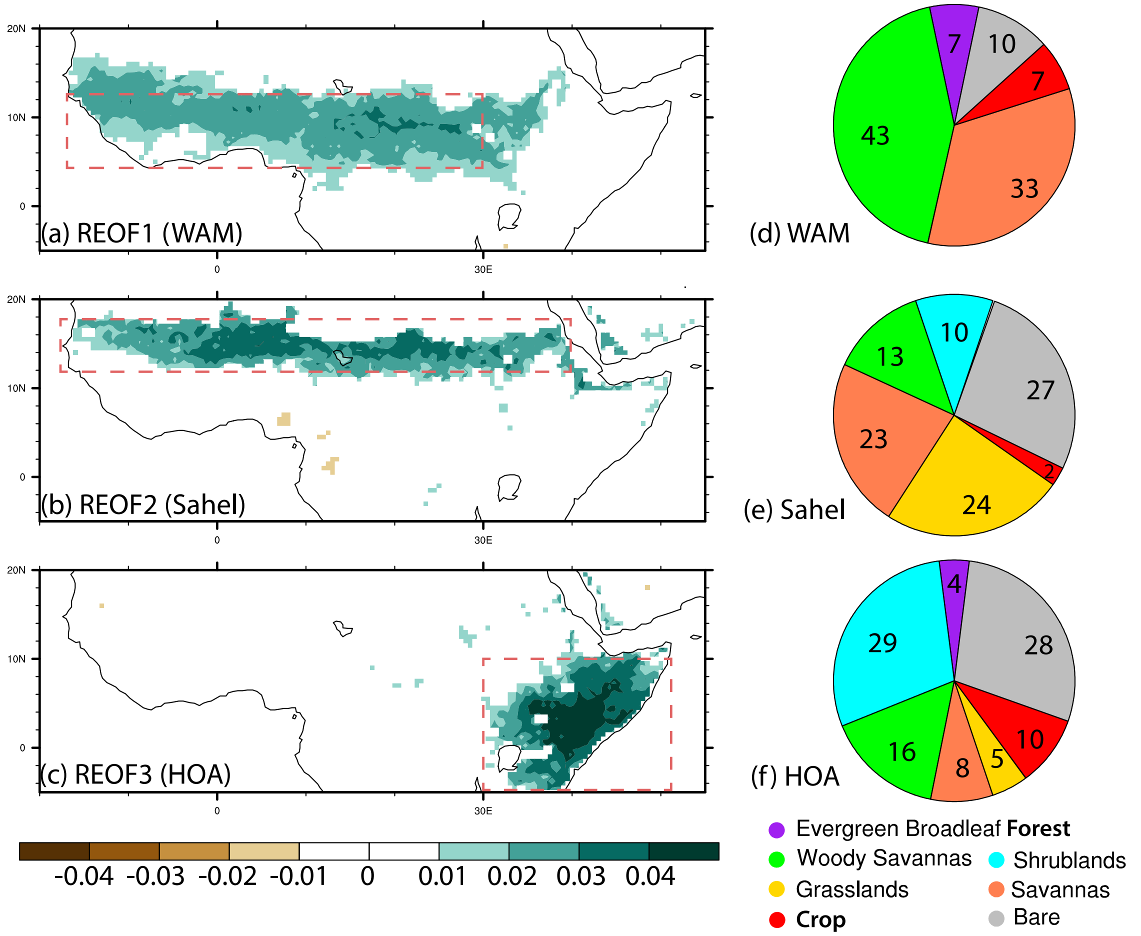

Supplementary Figure 8 | Spatial pattern of NDVI variability and distribution of land cover types across the West African Monsoon (WAM) region, Sahel, and Horn of Africa (HOA). Spatial pattern of the leading three rotated EOFs (REOF) in standardized NDVI, representing variability across the (a) broader WAM region (5˚N-12˚N, 20˚W-30˚E), (b) Sahel (12˚N-17˚N, 20˚W-40˚E), and (c) HOA (5˚S-10˚N, 30˚E-52˚E). Percent area of land cover types across the (d) WAM, (e) Sahel, and (f) HOA. Analysis is based on the Advanced Very High Resolution Radiometer (AVHRR) Global Inventory Modeling and Mapping Studies (GIMMS) NDVI3g dataset for 1982-2011[5]. The REOF analysis helps define regions (red boxes) for area-average NDVI to be included in the GEFA forcing matrix. The remotely sensed land cover data is from the International Satellite Land Surface Climatology Project (ISLSCP) initiative II International Geosphere-Biosphere Project (IGBP) DISCover and Simple Biosphere (SiB) Land Cover dataset.

Supplementary Table 1 | List of analyzed observational, remote sensing, and reanalysis datasets for each GEFA response variable. Asterisks denote datasets that incorporate remotely-sensed information.

Variables	Dataset	Analyzed Years	Spatial Resolution
Vertical motion, precipitable water, 2-m specific humidity, 10-m wind speed, sea-level pressure (SLP)	National Aeronautics and Space Administration (NASA) Modern-Era Retrospective Analysis for Research and Applications (MERRA)[6,*]	1982-2011	0.5° x 0.66°
	Japanese 55-year Reanalysis (JRA55)[7,*]	1982-2011	0.63° x 0.63°
	European Centre for Medium-Range Weather Forecast (ECMWF) Interim Reanalysis (ERA-In)[8,*]	1982-2011	0.75° x 0.75°
	National Centers for Environmental Prediction (NCEP)-Climate Forecast System Reanalysis (CFSR)[9,*]	1982-2010	0.5° x 0.5°
2-m air temperature	University of Delaware (UDEL) Terrestrial Air Temperature[10]	1982-2011	0.5° x 0.5°
	University of East Anglia Climatic Research Unit (CRU) Time Series (TS3.22)[11]	1982-2011	0.5° x 0.5°
Precipitation	UDEL Terrestrial Precipitation[10]	1982-2011	0.5° x 0.5°
	Global Precipitation Climatology Centre (GPCC)[12]	1982-2011	0.5° x 0.5°
	CRU TS3.22[11]	1982-2011	0.5° x 0.5°
	Global Precipitation Climatology	1982-2011	2.5° x 2.5°

	Project (GPCP)[13,*]		
	Climate Prediction Center (CPC) Merged Analysis of Precipitation (CMAP)[14,*]	1982-2011	2.5° x 2.5°
	Global Historical Climatology Network stations - daily	1982-2011	
Outgoing longwave radiation (OLR)	Advanced Very High Resolution (AVHRR) Pathfinder Atmospheres-Extended (PATMOS-x[15,*]	1982-2011	0.1° x 0.1°
	National Oceanic and Atmospheric Administration (NOAA) Climate Data Records (CDR)[16,*]	1982-2011	1° x 1°
Evapotranspiration (ET)	Mao's merged diagnostic ET product[17,*]	1982-2010	0.5° x 0.5°
	Global Land Evaporation Amsterdam Model (GLEAM) Global Evapotranspiration[18,*]	1982-2010	0.5° x 0.5°
Dust emission, concentration, and transport	MERRA-2[19,*]	1982-2011	0.5° x 0.63°
Dust frequency	Global Historical Climatology Network stations - hourly	1982-2011	

Supplementary Table 2 | Summary of multi-dataset evaluation and ranking. In order to reduce the observational error, weights are assigned to different data products according to their regional reliability when generating the multi-dataset PDF of the response. The listed weight rankings are specific to the Sahel region.

**Variable**	**Ranking criteria**	**Weight rankings**
SLP, 10-m wind, vertical motion	Evaluate surface u- and v-wind against data from 87 stations in terms of the	JRA55 > ERA-In > CFSR > MERRA

	temporal correlation and root-mean-square error	
Precipitation	Evaluate against GPCP satellite-gauge merged product and TRMM satellite product in terms of the temporal correlation, spatial correlation, and root-mean-square error	GPCP > GPCC = CMAP > UDEL> CRU
2-m air temperature	Number of Sahel stations included in dataset	UDEL > CRU
Precipitable water, 2-m specific humidity	Evaluate against NVAP precipitable water in terms of the temporal correlation	ERA-In > CFSR > JRA55 > MERRA
ET	Number of data sources included in dataset	Mao's ET > GLEAM
OLR	Number of included satellite instruments	PATMOS-x = CDR

Supplementary Table 3 | Evaluation of daily surface u- and v-wind from reanalyses against 87 stations across the Sahel (1979-2013): regional average temporal correlation (all p's < 0.1) and root-mean-square-error (RMSE, m s^{-1}) in daily u- and v-wind between station wind observations and the nearest grid cell from reanalysis. The determined weights are applied to SLP, 10-m wind speed, and vertical motion, since these variables are closely related to surface u- and v-wind.

**Variable**	**u-wind**		**v-wind**	
**Metric**	**Temp. Correlation**	**RMSE (m s^{-1})**	**Temp. Correlation**	**RMSE (m s^{-1})**
MERRA	0.53	2.39	0.55	2.56
CFSR	0.51	2.27	0.57	2.54
ERA-In	0.55	2.32	0.59	2.09
JRA-55	0.59	1.93	0.58	2.11

Supplementary Table 4 | Evaluation of daily precipitable water from reanalyses against NVAP (1988-2001) across the Sahel: regional average temporal correlation between daily NVAP and each reanalysis (all p's < 0.1). NVAP is chosen as a benchmark because of its spatial

and temporal coverage, especially over the Sahel region where radiosonde data is sparse. The determined weights are also applied to 2-m specific humidity, which is closely related to precipitable water.

Metric	Temp. Correlation*
MERRA	0.35
JRA-55	0.41
ERA-In	0.49
CFSR	0.46

*RMSE is not computed due to the wet bias in NVAP[20].

Supplementary Table 5 | Evaluation of monthly precipitation from gridded gauge observations against the satellite-gauge merged GPCP (1979-2014) across the Sahel: regional average temporal correlation (all p's < 0.1) and RMSE (cm mon^{-1}) between monthly precipitation anomalies from each precipitation dataset and GPCP. GPCP is considered as a benchmark because it incorporates both gauge observations and multiple remote-sensing products[13].

Metric	Temp. Correlation	RMSE (cm mon^{-1})
CRU	0.68	1.40
UDEL	0.72	1.32
CMAP	0.75	1.09
GPCC	0.79	1.18

Supplementary Table 6 | Evaluation of monthly precipitation from gridded observational datasets against TRMM multi-satellite precipitation analysis[21] (1999-2014) across the Sahel: regional average temporal correlation (all p's < 0.1), time-average spatial correlation (all p's < 0.1), and RMSE (cm mon^{-1}) between monthly precipitation anomalies from each precipitation dataset and TRMM.

	Temp. Correlation	Spat. Correlation	RMSE (cm mon^{-1})
CRU	0.47	0.81	1.58
UDEL	0.49	0.84	1.50

CMAP	0.57	0.87	1.43
GPCC	0.63	0.88	1.22
GPCP	0.67	0.92	0.91

Supplementary References

1. Lorenz, C., & Kunstmann, H. The hydrological cycle in three state-of-the-art reanalyses: Intercomparison and performance analysis. Journal of Hydrometeorology, 13 (5), 1397-1420 (2012).

2. Hewltson, B., Lennard, C., Nikulin, G., & Jones, C. CORDEX-Africa: a unique opportunity for science and capacity building. CLIVAR Exchanges, 17 (3), 6-7 (2012).

3. Jiang, Z., Wu, Y., Liu, Z., Wen, N., & Zhao, C. A diagnostic analysis of air temperature anomaly mode over China in 2009/2010 winter based on Generalized Equilibrium Feedback Assessment (GEFA) method. Journal of Tropical Meteorology, 21 (2), 121-130 (2015).

4. Rayner, N. A. et al. Global analyses of sea surface temperature, sea ice, and night marine air temperature since the late nineteenth century. Journal of Geophysical Research 108, 4407 (2003).

5. Zeng, F.W., Collatz, G. J., Pinzon J. E., & Ivanoff A. et al. Evaluating and quantifying the climate-driven interannual variability in Global Inventory Modeling and Mapping Studies (GIMMS) Normalized Difference Vegetation Index (NDVI3g) at global scales. Remote Sensing 5 (8), 3918-3950 (2013).

6. Rienecker, M. M. et al. MERRA: NASA's Modern-Era Retrospective Analysis for Research and Application. Journal of Climate 24, 3624-3648 (2011).

7. Kobayashi, S. et al. The JRA-55 Reanalysis: General specifications and basic characteristics. J. Meteor. Soc. Japan, 93 (1), 5-48 (2015).

8. Dee, D. P. et al. The ERA-Interim reanalysis: Configuration and performance of the data assimilation system, Quart. J. R. Meteorol. Soc. 137, 553-597 (2011).

9. Saha, S. et al. The NCEP Climate Forecast System Reanalysis. Bull. Amer. Meteor. Soc. 91 (8), 1015-1057 (2010).

10. Matsuura, K. & Willmott, C. J. Terrestrial precipitation: 1900-2008 gridded monthly time series (version 2.01) (2012) [Available online at http://climate.geog.udel.edu/~climate/html_pages/Global2_Ts_2009/README.global_p_ts_2009.html.]
11. Harris, I., Jones, P. D., Osborn, T. J. & Lister, D. H. Updated high-resolution grids of monthly climatic observation – the CRU TS3.10 dataset. Int. J. Climatol. 34 (3), 623-642 (2014).
12. Schneider, U., Fuchs, T., Meyer-Christoffer, A., & Rudolf, B. Global precipitation analysis products of the GPCC. Global Precipitation Climatology Centre (GPCC), DWD, Internet Publikation, 112 (2008).
13. Huffman, G. J. Estimates of root-mean-square random error for finite samples of estimated precipitation, J. Appl. Meteor. 36, 1191-1201 (1997).
14. Xie, S. P., & Arkin, P. A. Global precipitation: A 17-year monthly analysis based on gauge observations, satellite estimates, and numerical model outputs. Bull. Amer. Meteor. Soc. 78, 2539-2558 (1997).
15. Heidinger, A. K., Foster, M. J, Walther, A., & Zhao, X. The Pathfinder Atmospheres Extended (PATMOS-x) AVHRR climate dataset, Bull. Amer. Meteor. Soc. 95(6), 909-922 (2014).
16. National Research Council. Climate Data Records from environmental satellites: interim report. The National Academies Press, Washington, D. C., pp136 (2004).
17. Mao, J., Fu, W., Shi, X., Ricciuto, D. M., Fisher, J. B., Dickinson, R. E., & Schwalm, C. R. Disentangling climatic and anthropogenic controls on global terrestrial evapotranspiration trends. Environmental Research Letters 10 (9), 0094008 (2015).
18. Mu, Q., Heinsch, F. A., Zhao, M, & Running, S. W. Development of global evapotranspiration algorithm based on MODIS and global meteorology data. Remote Sensing of Environment 111, 519-536 (2007).
19. Bosilvich, M. G., Lucchesi, R., & Suarez, M. MERRA-2: File Specification, GMAO Office Note No. 9, http://gmao.gsfc.nasa.gov/pubs/docs/Bosilovich785.pdf (2015).
20. Amenu, G. G, & Kumar, P. NVAP and Reanalysis-2 global precipitable water products: Intercomparison and variability studies. Bull. Amer. Meteor. Soc 86 (2), 245 (2005).

21. Huffman, G.J. et al. The TRMM multisatellite precipitation analysis (TMPA): Quasi-global, multiyear, combined-sensor precipitation estimates at fine scales. Journal of Hydrometeorology, 8 (1), 38-55.
