# Supplementary material for: Observed positive vegetation-rainfall feedbacks in the Sahel dominated by a moisture recycling mechanism
Source: Nat Commun. 2017 Nov 30;8:1873. doi: 10.1038/s41467-017-02021-1 (PMC5707399; doi:10.1038/s41467-017-02021-1)
Supplement: Supplementary file 1 — Peer Review File [file 41467_2017_2021_MOESM1_ESM.pdf]

Editorial Note: this manuscript has been previously reviewed at another journal that is not operating a transparent peer review scheme. This document only contains reviewer comments and rebuttal letters for versions considered at Nature Communications.

Reviewer #1 (Remarks to the Author):

The review is attached.

Reviewer #2 (Remarks to the Author):

This is an interesting paper on the possible role of land surface-vegetation feedbacks in the sub-Sahelian West African monsoon region. I am returning the review early to allow the editors to, if necessary, seek additional reviews that can properly asses the sophisticated statistical methodology on which the results depend. I am not a skilled statistician and cannot do this myself. I will instead focus on the physical mechanisms.

Unlike prior efforts, the authors provide evidence that the land-vegetation-atmosphere feedback works via evapotranspiration (ET), and not via albedo, and is only in the late monsoon season with ocean forcing being dominant in the main monsoon season. These results are physically plausible and interesting.

The work is very impressive in its thoroughness. The authors have used essentially all available data and done their analysis using multiple combinations of data sets in order to improve robustness. I commend them for this since this is time consuming, difficult and requires great attention to details, and, in fact, is very rarely done in climate research. The technique used is well described but is totally unfamiliar to me. I have not met it before and have not read papers that make clear it works well or papers that criticize it so I cannot assess it.

Here are my concerns re the physical mechanisms. All these need to be answered and explained before publication is possible.

1. The argument seems to be that a wet monsoon encourages vegetation growth and higher ET that increases water recycling and hence precipitation. This effect occurs, reasonably, at the end of the monsoon season. What I don't get is why, at the end of the monsoon season, higher precipitation is associated with increased subsidence and higher sea level pressure! This should never happen.

2. Why is the ocean influence restricted to the main monsoon season? It states, lines 128-130, that teleconnections patterns are weaker in autumn than summer. The tropical teleconnection pattern primarily works via warming of the troposphere which is zonally uniform and should not depend on season. Also El Nino events typically strengthen from summer through fall and winter.

3. The mechanism should presumably mean that summers where ocean conditions favor a wetter monsoon should also mean these are longer monsoons as the proposed feedback comes into play to extend the monsoon season. Is this so? This addresses another point which is that it is required that the work go beyond a purely statistical approach to explain actually history and events in the West African monsoon, otherwise it will be of next to no interest to the general interest community.

Even if these changes are made, I wonder if the paper meets the high impact requirements of this journal. If they are fixed it would be an excellent fit for ERL and GRL, for example. I think ultimately it will depend on how the authors manage to link the sophisticated analysis back to actual West African Monsoon history and actual events of short/long, weak/failed monsoons and, hence, down to what really matters for people.

Reviewer #3 (Remarks to the Author):

What are the major claims of the paper?

The study claims to show observational evidence for a positive vegetation-rainfall feedback across the Sahel, confined to the post-monsoon season. It originates from moisture recycling mechanisms.

The authors conclude on the important role of vegetation in regulating atmosphere conditions and thereby potentially impacting hydrological extremes in the Sahel.

Are they novel and will they be of interest to others in the community and the wider field?

The results are of interest to the community. There is a widespread community that addresses the questions of vegetation-atmosphere feedbacks in the vulnerable region of the Sahel. The authors argue that novelty in their respective study comes in by the fact that they use different observational and reanalysis data sets and the GEFA method.

If the conclusions are not original, it would be helpful if you could provide relevant references.

The conclusions go along with similar studies, albeit specific details and some applied methods are indeed new. I would take the liberty to indicate latest own work (Klein et al. 2016 (Climate Dynamics, DOI 10.1007/s00382-016-3237-x) that investigated feedback between vegetation and rainfall and that was not cited.

Is the work convincing, and if not, what further evidence would be required to strengthen the conclusions?

While the authors stress that the observational evidence using the applied variety of methods and data sets is the highlight of the study, it is particularly the type of observations and data sets that has to be seen critically for the conclusions drawn: The crucial problem for the gridded data sets e.g. of precipitation and temperature is that the underlying number of stations has decreased dramatically in the last decades and years, particularly in Africa (Lorenz and Kunstmann, 2012, Journal of Hydrometeorology, DOI: http://dx.doi.org/10.1175/JHM-D-11-088.1). And this is the case for the variety of gridded precipitation and temperature data sets used in this study. In turn, this means that time series analyses of these products and respective conclusions on correlations with other variables (NDVI for example in this study) have to be done by considering these limitations. Real convincing observational evidence needs to consider original station values for precipitation or satellite derived information conditioned on the ground truth. The authors mention that they use GHCN station data for precipitation, but they use it only for wet day frequencies. The validation of the most crucial variable, i.e. precipitation, is done only in spatially aggregated manner for the entire region (table 5, page 29) and using only simplest performance measures (RMSE, temporal correlation). If observational evidence for such a complex interaction between precipitation and albedo should be convincing by purely statistical analyses, the study requires a spatially distributed approach, starting with the performance evaluation of the involved hydrometeorological variables and their spatial patterns. In this regard, authors need to consider satellite derived precipitation products like TRMM for example. Likewise, in times of increasing number of regional atmospheric models, the restriction to global reanalysis data, all in resolution larger than 0.5°, does not seem to be appropriate when considering the strong climate gradient in West Africa and the Sahel.

In addition, it is confusing to the reader that the real area of analysis is not shown. What exactly is the spatial extend of the domain for which Figures 1 ,2, 3 and extended data tables 3,4, and 5, refer to? In addition, it is confusing to read several times that the analysis was done over North Africa (line 113, 114, 219, 338, etc.); also GEFA is applied over North Africa, but the study deals with the Sahel, which is clearly Sub-Saharan Africa. It is likewise confusing that in Extended Data Figure 2 the Horn of Africa is addressed. In all Figures (1, 2, 3, 4, etc.), the time period of the performed statistical analysis is missing. One needs to assume it is 1982-2011, as extended Data Table indicates this period.

Will the paper will influence thinking in the field?

It will be a further contribution among others.

Comment on the appropriateness and validity of the statistical analysis, as well the ability of a researcher to reproduce the work, given the level of detail provided.

Two non-published articles about the GEFA method and more details are in the additional material. These articles deal with North Africa. As they are not published they cannot be cited and cannot be used in any argumentation to my opinion.

Thank you for your useful comments. We responded to your comments and incorporated the suggested revisions to our manuscript.

Reviewer #1

Overall:
This study applied the multi-observed statistical approaches to address the question of which physical mechanism is dominant in the positive vegetation and rainfall feedbacks in the Sahel. They found that moisture recycling mechanism is dominant in the positive feedback in the Sahel, compared to both the albedo and roughness mechanisms, on the seasonal to interannual time scales. Additionally, vegetation-dust feedback was observed as a potential mechanism for the positive vegetation-rainfall feedback. The previous studies have identified the vegetation and rainfall feedbacks based on largely model simulations and relatively less observational analysis. This study robustly quantified the vegetation-climate feedbacks by "assessing the uncertainty in various observational products", weighted by the estimated regional reliability in North Africa. The main statistical method used in this study (Generalized Equilibrium Feedback Assessment, GEFA) could contribute to "robustness in identifying the observed signal of vegetation forcing on climate" from the atmospheric internal noise and oceanic forcing, which was limited in the univariate or multivariate linear regression-based methods in the previous studies. The results from the multi-observed statistical analysis identified "moisture recycling mechanism is dominant" among the underlying physical mechanisms (moisture, albedo and roughness mechanisms) for the positive vegetation-rainfall feedbacks in the Sahel. Based on the methodological approaches and scientific merits, this study is novel and will be of great interest to climate as well as ecology communities. This is novel and well-written manuscript based on the scientific logic and merit and statistical robustness. I think this study could make an advance in biosphere and atmosphere interaction study by enhancing the knowledges on biophysical processes in the coupled Earth system. The multi-observed empirical assessment of the feedback mechanism by using GEFA could be important benchmark for the observational study in climate science. Therefore, I recommend the publication of this study after revising the manuscript with the specific comments suggested below.

Specific/editorial comments:
1) Observational quantification of the relative contribution between oceanic vs. terrestrial drivers: This study has applied more robust statistics to quantify the relative importance of ocean (SST) and land (vegetation) forcings on the Sahel precipitation. However, one of the previous study cited by this study (He and Lee, 2016) has assessed the relative associations of vegetation and SST in explaining the summer rainfall in the Sahel by using Granger causality analysis. Thus, the related parts in manuscript should be

revised to recognize the previous work and, more importantly, to stress out the statistical robustness of this study. I found the related sentences in L16-19 ("nor its relative importance"), L73-77 ("do not tease out potential oceanic impacts"), and L91-93 ("has never been observational quantification").

The text was revised on line 69: "do not fully tease out", and line 87 ("there has never been rigorous observational quantification") in the revised manuscript to recognize the previous ocean versus land impacts by the Granger causality analysis (He and Lee, 2016). In addition, we have emphasized the limitation in the previous analysis on lines 89-93 of the revised manuscript: "Although the multiple regression-based study attempted to quantify the relative contribution of variability in Atlantic SST and Sahel NDVI to Sahel rainfall variability[25], the results are uncertain due to the limited reliability of multiple regression in separating impacts from individual forcings and are not directly comparable with previous modeling studies due to the absence of remote oceanic impacts assessed in their study." We also stress the statistical robustness of the current study on lines 168-174 of the revised manuscript: "In contrast to the previous observational study based on Granger causality analysis[25], the current GEFA-based analysis includes both nearby and remote oceanic forcings and considers multiple observational datasets, thereby providing a more convincing quantification of oceanic versus terrestrial contributions to Sahel rainfall variability. Furthermore, by considering both nearby and remote oceanic impacts, the GEFA-based assessment of oceanic versus terrestrial contributions is directly comparable with previous modeling studies that modify global SSTs through dynamical experiments[10,12]."

2) Different numbers of forcing matrix between ocean and terrestrial forcings: "According to GEFA, oceanic forcings largely overwhelm terrestrial forcings on the Sahel's precipitation and temperature… (L115-116)". I wonder if the result is related to the different numbers of forcing matrix (17 SST and 3 NDVI fields). If so, the numbers of forcing matrix should be adjusted by selecting the oceanic candidates based on statistical significance and physical importance for the climate in the Sahel.

The impact of an imbalanced forcing matrix, with more oceanic forcings than land forcings, is not likely a significant reason for the dominant oceanic impacts, because the imbalance has been largely reduced by applying stepwise GEFA. Indeed, the number of selected oceanic forcings varies from 1-5 by season, compared with 0-3 of land forcings, for Sahel rainfall. Therefore, the difference between the actual number of oceanic and land forcings assessed in stepwise GEFA is not that large.

To further test the potential impact of imbalanced oceanic versus terrestrial forcings on the estimated explained variance, we expanded the number of NDVI forcings by dividing the Sahel region into four sub-regions so that the total number of NDVI forcings is six (four sub-Sahel regions, WAM, and HOA). But the explained variance in Sahel rainfall and 2-m air temperature by ocean versus land forcings does not change significantly (Figure R1a,b). Indeed, the number of oceanic versus terrestrial forcings selected does not change significantly in the test case. Another test is done to further expand the terrestrial forcings by including 11 remote NDVI regions in central and south Africa (17 NDVI forcings versus 17 SST forcings, Figure R1c,d), but the explained variance does not change significantly from Figure 1. A similar test is done by

including the leading 3 SST EOFs in the forcing matrix (25 SST and 3 NDVI forcings, Figure R1e,f). The result is again almost identical to that in Figure 1. Note that the multi-dataset uncertainty in explained variance in these tests is larger than that in Figure 1, mainly due to the decreased reliability of GEFA with increased number of forcings in the forcing matrix (Yu et al. 2017). These tests further ensure that the explained variance result is not likely affected by the different number of ocean versus land forcings. We have clarified this point on lines 156-158 of the revised manuscript: "Note that the apparent greater contribution from oceanic forcings is not an artifact of the imbalanced number of oceanic versus terrestrial forcings, as confirmed by additional sensitivity tests that modulate the number of either type of forcing."

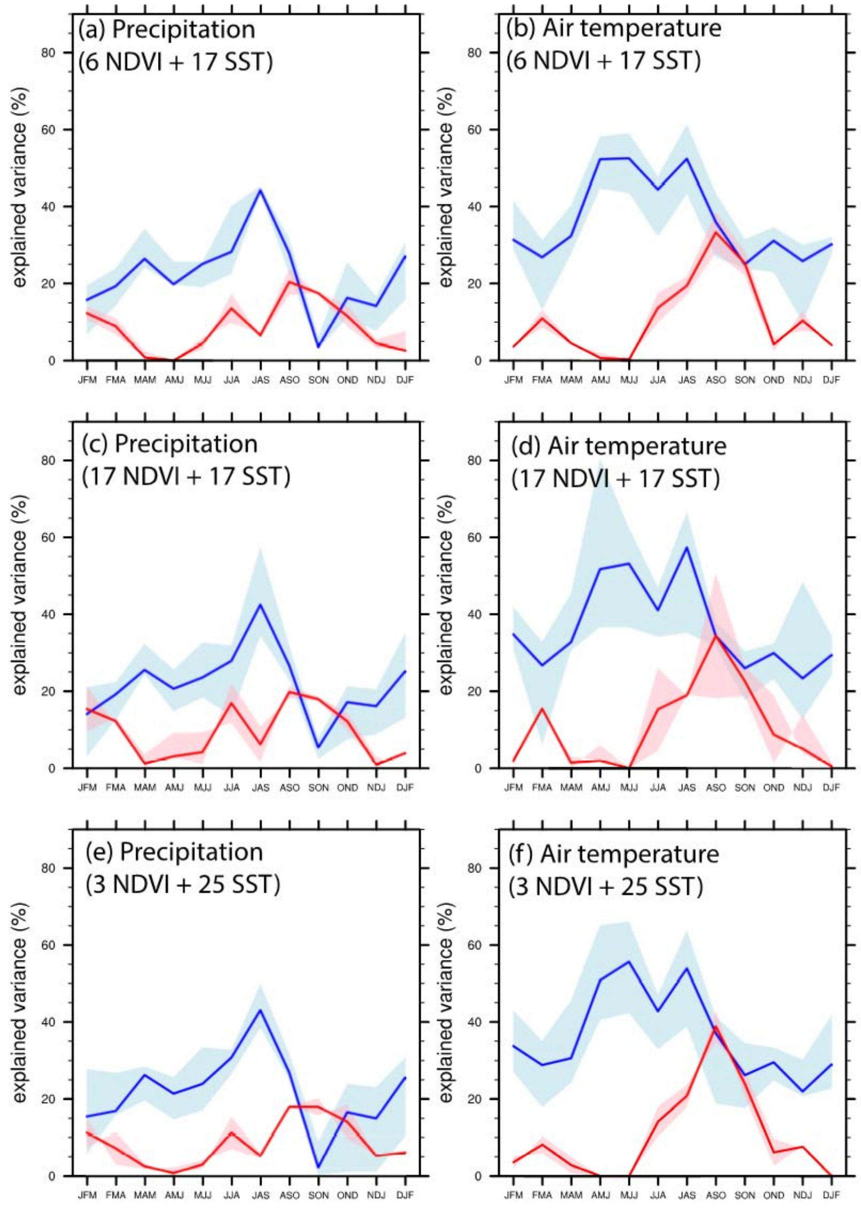

Figure R1 Same as Figure 1 but with (a, b) 6 NDVI + 17 SST forcings, (c, d) 17 NDVI + 17 SST, or (e, f) 3 NDVI + 25 SST forcings, compared with 3 NDVI + 17 SST forcings in Figure 1.

3) L45-50, p. 2: The following would be one of important references for the positive vegetation-rainfall feedback in the Sahel.
Foley, J. A., Coe, M. T., Scheffer, M., & Wang, G. (2003). Regime shifts in the Sahara and Sahel: Interactions between ecological and climatic systems in Northern Africa. Ecosystems, 6, 524–532.10.1007/s10021-002-0227-0.
Added on lines 30 and 176 and the reference list.

4) L182, p. 6: Add “(Figure 3b)” after …of the Sahel.
Done on line 224.

5) L191-196, p. 7: Mention the associated figures in the appropriate place in the sentences to support the results.
Done on line 235.

6) L205, p. 7: Mention the related figure (Figure 4a) after …one standard deviation.
Done on line 251.

7) L206, p. 7: Change “southward” to “southwestward”.
Done on line 252.

Reviewer #2 (Remarks to the Author):

This is an interesting paper on the possible role of land surface-vegetation feedbacks in the sub-Sahelian West African monsoon region. I am returning the review early to allow the editors to, if necessary, seek additional reviews that can properly asses the sophisticated statistical methodology on which the results depend. I am not a skilled statistician and cannot do this myself. I will instead focus on the physical mechanisms.

Unlike prior efforts, the authors provide evidence that the land-vegetation-atmosphere feedback works via evapotranspiration (ET), and not via albedo, and is only in the late monsoon season with ocean forcing being dominant in the main monsoon season. These results are physically plausible and interesting.

The work is very impressive in its thoroughness. The authors have used essentially all available data and done their analysis using multiple combinations of data sets in order to improve robustness. I commend them for this since this is time consuming, difficult and requires great attention to details, and, in fact, is very rarely done in climate research. The technique used is well described but is totally unfamiliar to me. I have not met it before and have not read papers that make clear it works well or papers that criticize it so I cannot assess it.

Here are my concerns re the physical mechanisms. All these need to be answered and explained before publication is possible.

1. The argument seems to be that a wet monsoon encourages vegetation growth and higher ET that increases water recycling and hence precipitation. This effect occurs, reasonably, at the end of the monsoon season. What I don't get is why, at the end of the monsoon season, higher precipitation is associated with increased subsidence and higher sea level pressure! This should never happen.

According to our observational analysis and previous modeling studies (Charney 1975; Zeng et al. 1999; Wang et al. 2004), anomalies in Sahel vegetation cover trigger multiple simultaneous feedbacks such as moisture recycling, albedo feedback, momentum feedback, etc, each with their own strength and competing with each other. Here, the dynamic response of greater stability in response to positive NDVI anomalies is swamped by the moisture recycling feedback. We have clarified this point on lines 220-222 of the revised manuscript: "The increased atmospheric stability in response to positive NDVI anomalies, although swamped by the moisture recycling mechanism, is counter to the dynamic responses expected through the classic vegetation-albedo feedback mechanism."

We think that higher precipitation is not a result of increased subsidence and higher sea-level pressure, but they are all results of increased ET during the late-to-post monsoon season in Sahel. Higher precipitation is caused by the enhanced moisture recycling associated with increased ET in response to increased vegetation cover and soil moisture (Figure 2). On the other hand, increased ET causes a change in Bowen ratio, leading to low-level cooling (Figure 3a), decreased lapse rate, and low-level stabilization of the atmosphere (Supplementary Figure 6).

One might be concerned that at the end of the monsoon season, the mean climatology is subsidence on the monthly average. But convection can still happen on some days or at least some time during the day, when an increase in the total precipitable water as part of the enhanced moisture recycling in response to positive NDVI anomalies can lead to increased precipitation.

2. Why is the ocean influence restricted to the main monsoon season? It states, lines 128-130, that teleconnections patterns are weaker in autumn than summer. The tropical teleconnection pattern primarily works via warming of the troposphere which is zonally uniform and should not depend on season. Also El Nino events typically strengthen from summer through fall and winter.

The seasonality of oceanic influence on the Sahel regional climate is also presented for ET, precipitable water, and 850-hPa vertical motion (Figure R2). The oceanic influence on ET, precipitable water, and 850-hPa vertical motion are also stronger during the main monsoon season than the post-monsoon season.

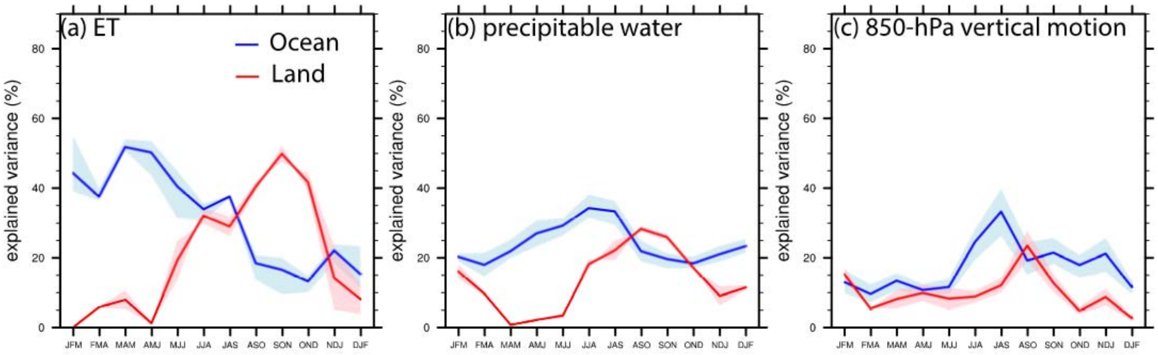

Figure R2 Same as Figure 1 but for (a) ET, (b) precipitable water, and (c) 850-hPa vertical motion.

The seasonality of the oceanic influence on Sahel regional climate may come from multiple aspects. One source of the seasonality of oceanic impacts comes from the seasonality of tropical Atlantic SSTs, one of the most important SST forcings for the Sahel. The zonal SST gradient in the Atlantic Niño mode (first EOF in tropical Atlantic SST, TA1) maximizes during the peak monsoon season (Latif and Grotzner 2001; Figure R3). Since we use seasonal SST EOFs in the forcing matrix, the seasonality of the TA1 forcing pattern affects the influence of TA1 on Sahel rainfall, supporting larger oceanic impacts on the Sahel rainfall in the monsoon season.

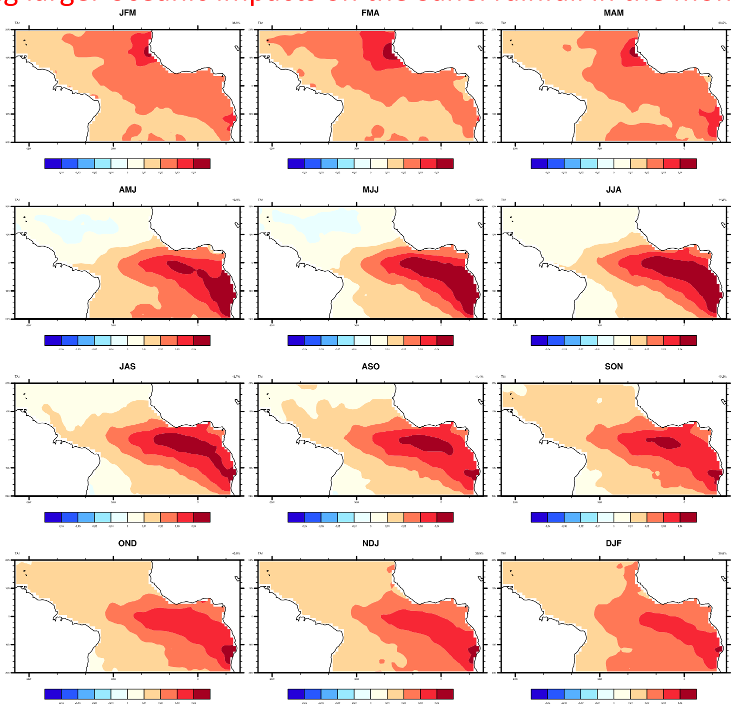

Figure R3 Spatial pattern (unitless) of first EOF in tropical Atlantic SST in each season.

In terms of ENSO, Joly and Voldoire (2009) also reported that in observations, the influence of ENSO on the West African monsoon (WAM) peaks during the developing phase of El Niño/La Niña or during the decay of some long-lasting La Niña events. In the El Niño

developing phase, the warm SST anomalies move westward over the equatorial Pacific, leading to strong subsidence over the western Sahel as a stationary Kelvin wave response originating from the East Pacific (Rowell 2001). The observed seasonality in impacts of the tropical Pacific SSTs on the WAM is confirmed by sensitivity experiments that modify regional SSTs in atmospheric general circulation models (Mohino et al. 2011). Since the current paper focuses on vegetation feedbacks, we did not include detailed discussion on the influence of ENSO or Atlantic Niño on Sahel rainfall, but referred to relevant papers on line 152 of the revised manuscript.

Another potential source of the seasonality of oceanic influence comes from the significance test in the GEFA method, related to the seasonality of Sahel rainfall variability. In the dry season (October-April), limited interannual variability in Sahel rainfall, especially less normality in the PDF of the anomalies, tend to lower the accuracy of the Monte Carlo bootstrapping testing (Efron and Efron, 1982), leading to less reliable significance test results than in the monsoon season when the precipitation distribution is more normal (Figure R4).

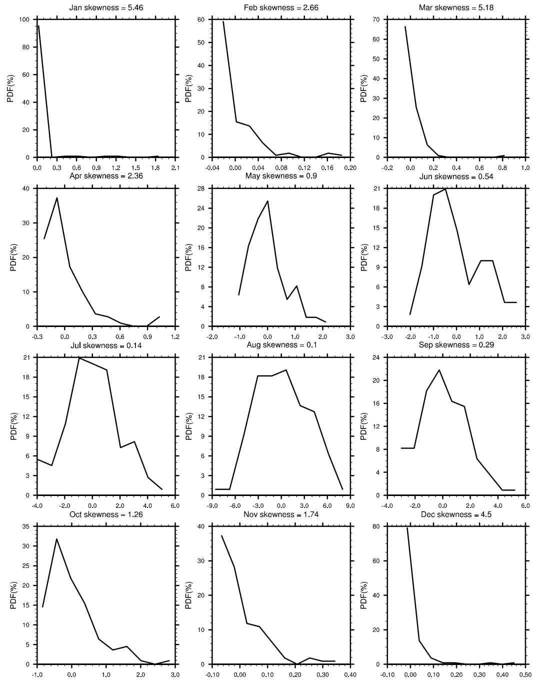

Figure R4 Probability distribution function of Sahel precipitation anomalies (cm month^{-1}) (1982-2011, from GPCP) in each month.

3. The mechanism should presumably mean that summers where ocean conditions favor a wetter monsoon should also mean these are longer monsoons as the proposed feedback comes into play to extend the monsoon season. Is this so?

The temporal correlation between July-August rainfall and September rainfall is 0.58 during 1901-2014 based on detrended GPCC precipitation data, supporting the hypothesis that wetter monsoons driven by oceanic forcings tend to be longer with vegetation's regulation. This is stated in the revised manuscript on lines 206-212: "The identified positive vegetation-rainfall feedback in the Sahel during the late-to-post monsoon season suggests that an anomalous wet

monsoon tends to persist longer, since enhanced vegetation growth and wet soil associated with anomalously abundant rainfall likely cause an extended monsoon season over the Sahel. This proposed link between an anomalously wet and extended monsoon season is supported by a temporal correlation of 0.58 between the observed detrended JJA rainfall and September rainfall anomalies over the Sahel during 1901-2014." In addition, the probability of a subsequent wet (dry) September following a wet (dry) July-August is 80% (75%) in the Sahel, where wet (dry) is defined when the rainfall anomaly exceeds positive (negative) one interannual standard deviation.

This addresses another point which is that it is required that the work go beyond a purely statistical approach to explain actually history and events in the West African monsoon, otherwise it will be of next to no interest to the general interest community. Even if these changes are made, I wonder if the paper meets the high impact requirements of this journal. If they are fixed it would be an excellent fit for ERL and GRL, for example. I think ultimately it will depend on how the authors manage to link the sophisticated analysis back to actual West African Monsoon history and actual events of short/long, weak/failed monsoons and, hence, down to what really matters for people.

In order to link our statistical analysis back to the actual West African Monsoon history, we use the GEFA feedback coefficients associated with oceanic and terrestrial forcings, respectively, to build prediction models for the Sahel rainfall during the peak monsoon and late-to-post monsoon seasons (Supplementary Figure 3). Results are discussed on lines 130-139 of the revised manuscript: "Indeed, Sahel rainfall variability during SON of 1982-2010 is largely successfully reconstructed by the GEFA-based prediction model containing only terrestrial NDVI forcings, with a temporal correlation of 0.79 between the multi-dataset average predicted and observed time series, compared with that of 0.81 using both SST and NDVI forcings, and 0.56 using only SST forcings (Supplementary Figure 3b). In contrast to the late-to-post monsoon season and consistent with previous model-based findings[10,12], the monsoon rainfall over the Sahel during June-August is mostly regulated by oceanic drivers with a temporal correlation between the predicted and observed rainfall of 0.84 using only oceanic forcings, compare with 0.88 using both oceanic and terrestrial forcings (Supplementary Figure 3a). "

Using GEFA, we are able to decompose the contribution from each oceanic and terrestrial forcings on individual drought and pluvial cases, as explained on lines 139-148 of the revised manuscript: "The contribution from each oceanic and terrestrial forcing on individual drought and pluvial cases can be decomposed by GEFA. For example, during 1984 (Supplementary Figure 4a,b), which was the driest year in the Sahel during 1982-2011, an anomalously dry monsoon was favored by the Atlantic and Indian SST anomalies, as well as anomalously sparse vegetation cover in the Sahel, West African monsoon region, and Horn of Africa. This drought continued during the post-monsoon season, mainly driven by the anomalies in Sahel vegetation cover. As another example, during 1999 (Supplementary Figure 4c,d), which was one of the wettest years during the study period, an anomalously wet Sahel monsoon was mainly supported by La Niña conditions, and extended into the post-monsoon season, when oceanic impacts largely diminished and terrestrial forcings were allowed to dominate. "

Reviewer #3 (Remarks to the Author):

What are the major claims of the paper?
The study claims to show observational evidence for a positive vegetation-rainfall feedback across the Sahel, confined to the post-monsoon season. It originates from moisture recycling mechanisms. The authors conclude on the important role of vegetation in regulating atmosphere conditions and thereby potentially impacting hydrological extremes in the Sahel.

Are they novel and will they be of interest to others in the community and the wider field?
The results are of interest to the community. There is a widespread community that addresses the questions of vegetation-atmosphere feedbacks in the vulnerable region of the Sahel. The authors argue that novelty in their respective study comes in by the fact that they use different observational and reanalysis data sets and the GEFA method.

If the conclusions are not original, it would be helpful if you could provide relevant references.
The conclusions go along with similar studies, albeit specific details and some applied methods are indeed new. I would take the liberty to indicate latest own work (Klein et al. 2016 (Climate Dynamics, DOI 10.1007/s00382-016-3237-x) that investigated feedback between vegetation and rainfall and that was not cited.

We emphasize the novelty in our study on lines 262-267 in the revised manuscript: "The current study presents the first convincing observational evidence for the model-hypothesized positive vegetation-rainfall feedbacks in the Sahel, by successfully isolating terrestrial feedbacks from oceanic drivers and systematically examining multiple observational datasets in order to quantify observational uncertainty in feedback response estimates. The identification of key oceanic and terrestrial drivers will aid in successful seasonal predictions of regional climate in Sahel, a region that is highly vulnerable to hydrological extremes."

Thanks for pointing out the useful reference. It is cited on lines 214-220: "Low-level cooling (due to increased ET and latent heat flux and thus decreased Bowen ratio), weakened lapse rate (Figure 3a), higher surface pressure, and anomalous lower- to mid-tropospheric subsidence (Supplementary Figure 5) in response to positive NDVI anomalies support an active stability mechanism, consistent with findings in the regional modeling study regarding the impacts of vegetation cover on heat and moisture fluxes over the Sahel and West African monsoon region[41]." in the revised manuscript.

Is the work convincing, and if not, what further evidence would be required to strengthen the conclusions?
While the authors stress that the observational evidence using the applied variety of methods and data sets is the highlight of the study, it is particularly the type of observations and data sets that has to be seen critically for the conclusions drawn: The crucial problem for the gridded data sets e.g. of precipitation and temperature is that the underlying number of stations has decreased dramatically in the last decades and years, particularly in Africa (Lorenz and Kunstmann, 2012, Journal of Hydrometeorology, DOI: http://dx.doi.org/10.1175/JHM-D-11-

088.1). And this is the case for the variety of gridded precipitation and temperature data sets used in this study. In turn, this means that time series analyses of these products and respective conclusions on correlations with other variables (NDVI for example in this study) have to be done by considering these limitations. Real convincing observational evidence needs to consider original station values for precipitation or satellite derived information conditioned on the ground truth. The authors mention that they use GHCN station data for precipitation, but they use it only for wet day frequencies. The validation of the most crucial variable, i.e. precipitation, is done only in spatially aggregated manner for the entire region (table 5, page 29) and using only simplest performance measures (RMSE, temporal correlation). If observational evidence for such a complex interaction between precipitation and albedo should be convincing by purely statistical analyses, the study requires a spatially distributed approach, starting with the performance evaluation of the involved hydrometeorological variables and their spatial patterns. In this regard, authors need to consider satellite derived precipitation products like TRMM for example.
Likewise, in times of increasing number of regional atmospheric models, the restriction to global reanalysis data, all in resolution larger than 0.5°, does not seem to be appropriate when considering the strong climate gradient in West Africa and the Sahel.

We did not use TRMM directly in our analysis because TRMM is too short for applying GEFA, as concluded by our GEFA validation paper (Yu et al. 2017). However, the precipitation products are evaluated against TRMM in terms of the regional average temporal correlation, temporal average spatial correlation, and RMSE (Supplementary Table 6 in the revised manuscript). Evaluation against TRMM leads to the same conclusion as the evaluation against GPCP about the relative reliability of these products, that is GPCP>GPCC>CMAP>UDEL>CRU. This is because GPCP also includes satellite precipitation measurements from TRMM.

Instead of the short TRMM, we analyzed the long, satellite-based Tropical Application of Meteorology isomg SATellite and ground based observation (TAMSAT) rainfall dataset (Maidment et al. 2017) during 1983-2011. The regional average precipitation response to local NDVI anomalies in Sahel based on TAMSAT is largely consistent with that presented in the main text in terms of both sign and magnitude (Figure R5a). Beyond the regional average response, we also examined the spatial pattern (Figure R5b). Positive vegetation-rainfall feedbacks are identified across the majority of the Sahel, while the response magnitude maximizes in the climatologically wetter and more densely vegetated regions.

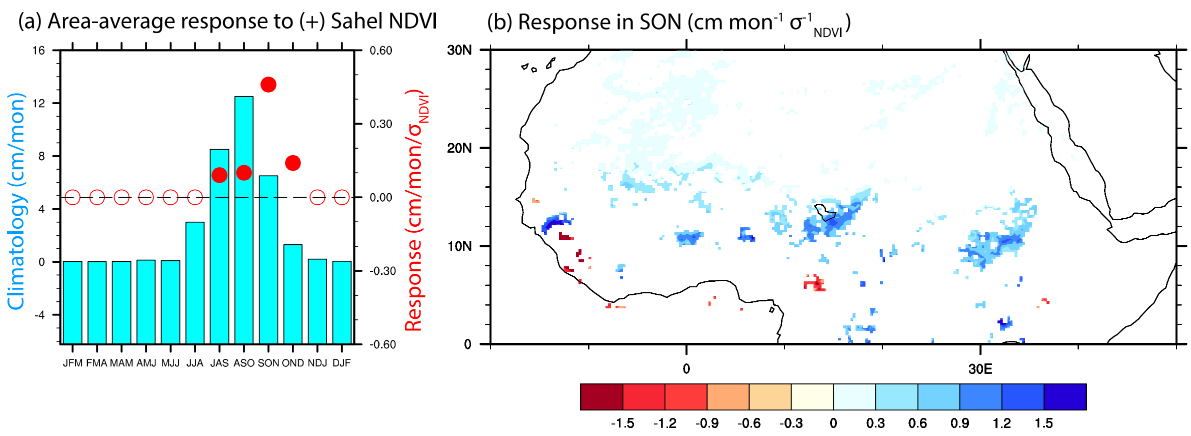

Figure R5 Precipitation response to positive Sahel NDVI anomalies based on the satellite-based TAMSAT precipitation estimates during 1983-2011. (a) Seasonal cycle of area-average Sahel precipitation response, with similar figure elements as Figure 2f. (b) Spatial pattern in precipitation response in SON. Only statistical significant ($p<0.1$) responses are shown in b, according to the Monte Carlo bootstrap test.

Beyond the gridded precipitation and air temperature products and reanalyses, we also examined the GEFA response for precipitation, 2-m air temperature, and 10-m wind speed from station observations (Supplementary Figure 7 in the revised manuscript). Although we are not able to repeat the GEFA work with all of the variables from real observations instead of reanalysis, these three variables represent the main conclusions and mechanisms discussed in this paper: precipitation directly refers to the vegetation-rainfall feedback, 2-m air temperature reflects albedo feedback mechanism, and 10-m wind speed represents momentum feedback mechanism. The responses are very similar between the gridded products and station observation in these three variables, confirming the findings presented in this paper.

In order to address the declining number of stations during the study period, we repeated the analysis in Supplementary Figure 6 with stations that have more than 20 years of data (Figure R6). The assessed feedbacks are very similar to those using all available stations, indicating minimum impacts of the declining number of stations on the results.

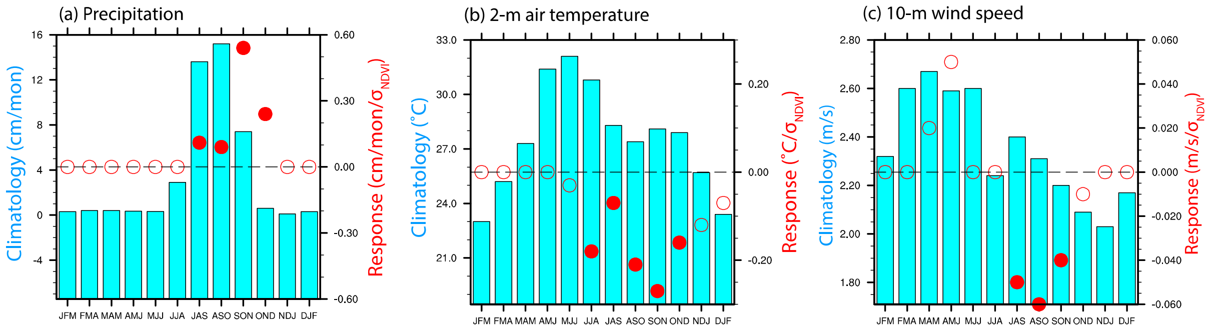

Figure R6 Observed response to positive local NDVI anomalies across the Sahel based on weather stations that have more than 20 years of data: (a) precipitation, (b) 2-m air temperature, and (c) 10-m wind speed. Figure elements are similar to Figure 2 (f).

In terms of the potential influence of coarse global reanalyses on the results, we analyzed the regional downscaling of ERA-Interim from CORDEX-Africa (Supplementary Data 1 and Supplementary Figure 8) in precipitation, surface air temperature, surface wind speed, and sea-level pressure. The results are largely consistent with those presented in the main text using gridded observations and global reanalyses.

Reference: Maidment, R.I. et al. A new, long-term daily satellite-based rainfall dataset for operational monitoring in Africa. Nature Scientific Data, 4 (170063) (2017).

In addition, it is confusing to the reader that the real area of analysis is not shown. What exactly is the spatial extend of the domain for which Figures 1 ,2, 3 and extended data tables 3,4, and 5, refer to? In addition, it is confusing to read several times that the analysis was done over North Africa (line 113, 114, 219, 338, etc.); also GEFA is applied over North Africa, but the study deals with the Sahel, which is clearly Sub-Saharan Africa. It is likewise confusing that in Extended Data Figure 2 the Horn of Africa is addressed. In all Figures (1, 2, 3, 4, etc.), the time period of the performed statistical analysis is missing. One needs to assume it is 1982-2011, as extended Data Table indicates this period.
The geographic boundary of Sahel and the time period of the analysis in this study are provided on lines 346-348 of the revised manuscript: "Regional average responses are obtained by applying GEFA to the atmospheric fields averaged across the Sahel (12˚N-17˚N, 20˚W-40˚E). The analysis is performed for 1982-2011." A map showing the Sahel region is added to Figure 1a. The time period is added to all figure captions. We have reworded geographic descriptions on lines 113 ("Sahel"), line 114 ("Sahel"), line 248-249 ("across the southern boundary of North Africa's major dust source regions within the Sahel"), and Supplementary Table 2 of the revised manuscript to make it clear that the analysis focuses on Sahel.

Will the paper will influence thinking in the field?
It will be a further contribution among others.

Comment on the appropriateness and validity of the statistical analysis, as well the ability of a researcher to reproduce the work, given the level of detail provided.
Two non-published articles about the GEFA method and more details are in the additional material. These articles deal with North Africa. As they are not published they cannot be cited and cannot be used in any argumentation to my opinion.
One of the GEFA method papers (Wang et al. 2017) has now been published. We have updated the reference in the revised manuscript. A revision of the other GEFA method paper (Yu et al. 2017) is in review with Journal of Climate.

Reviewer #1 (Remarks to the Author):

The authors have made effort to improve this manuscript according to the specific and minor comments which were suggested. I therefore recommend its publication.

Reviewer #2 (Remarks to the Author):

The authors have done an excellent job modifying the manuscript in response to my review and the questions I had are now answered well and concisely in the main manuscript text.
My only remaining question concerns the comparison of the observed time histories of Sahel and the statistical modeled histories. Typically when we do that with regression techniques we develop the model on a subset of the data and test against independent data (e.g. develop on recent years and test with earlier years). If the data are short a different approach would be a "leave on out" method where you develop the model withholding one data point and then predict that one and build up a predicted time history one at a time.
What did the authors do using their fancy statistical method?

Reviewer #4 (Remarks to the Author):

The paper clarifies the nature of the vegetation-rainfall feedback in the Sahel region using a new and powerful statistical method referred as GEFA. It is therefore of high interest for our community. I think previous comments from reviewer 3 were properly addressed. The only drawback I see is that the oceanic and terrestrial drivers considered in the GEFA analysis just explain half of the variance in observed Sahel precipitation and air temperature. This suggests that other factors not taken into account in the analysis may be more significant, leading to a different mechanism as that proposed by the authors. Nevertheless, I feel this limitation is properly explained in the paper. I therefore recommend this paper be accepted for publication.

Dear Dr. Tynan and reviewers,

Thank you for the valuable suggestions and comments. We have made associated revisions in our manuscript.

Yan

REVIEWERS' COMMENTS:

Reviewer #1 (Remarks to the Author):

The authors have made effort to improve this manuscript according to the specific and minor comments which were suggested. I therefore recommend its publication.
Thank you.

Reviewer #2 (Remarks to the Author):

The authors have done an excellent job modifying the manuscript in response to my review and the questions I had are now answered well and concisely in the main manuscript text.
My only remaining question concerns the comparison of the observed time histories of Sahel and the statistical modeled histories. Typically when we do that with regression techniques we develop the model on a subset of the data and test against independent data (e.g. develop on recent years and test with earlier years). If the data are short a different approach would be a "leave on out" method where you develop the model withholding one data point and then predict that one and build up a predicted time history one at a time.
What did the authors do using their fancy statistical method?
Thanks for the suggestion. The predicted time series presented in Supplementary Figure 1 are actually obtained through the "leave one out" approach. We have clarified this in the caption of Supplementary Figure 1:" The predicted time series are obtained through the leave-one-out approach, in which the GEFA feedback matrix is estimated using all years' data except the targeting one."

Reviewer #4 (Remarks to the Author):

The paper clarifies the nature of the vegetation-rainfall feedback in the Sahel region using a new and powerful statistical method referred as GEFA. It is therefore of high interest for our community. I think previous comments from reviewer 3 were properly addressed. The only drawback I see is that the oceanic and terrestrial drivers considered in the GEFA analysis just explain half of the variance in observed Sahel precipitation and air temperature. This suggests that other factors not taken into account in the analysis may be more significant, leading to a different mechanism as that proposed by the authors. Nevertheless, I feel this limitation is properly explained in the paper. I therefore recommend this paper be accepted for publication.
Thank you.
